# Marine Antimicrobial Peptide as a Promising Alternative to Polymyxin B

**DOI:** 10.3390/md24050154

**Published:** 2026-04-27

**Authors:** Victoria N. Safronova, Vladislav A. Lushpa, Victoria O. Shipunova, Marta V. Volovik, Kira L. Dobrochaeva, Roman N. Kruglikov, Ilia A. Bolosov, Dmitrii E. Dashevskii, Alexey V. Mishin, Oleg V. Batishchev, Olga V. Korobova, Alexander I. Borzilov, Gulsara A. Slashcheva, Igor A. Dyachenko, Eduard V. Bocharov, Pavel V. Panteleev, Tatiana V. Ovchinnikova

**Affiliations:** 1 M.M. Shemyakin & Yu.A. Ovchinnikov Institute of Bioorganic Chemistry, Russian Academy of Sciences, 117997 Moscow, Russia; victoria.saf@ibch.ru (V.N.S.); lushpa1696@gmail.com (V.A.L.); viktoriya.shipunova@phystech.edu (V.O.S.); kira.dobrochaeva@gmail.com (K.L.D.); kruglikov1911@mail.ru (R.N.K.); bolosov@ibch.ru (I.A.B.); edvbon@mail.ru (E.V.B.); p.v.panteleev@gmail.com (P.V.P.); 2 Moscow Center for Advanced Studies, 123592 Moscow, Russia; flyhawkdim@gmail.com (D.E.D.); mishinalexej@gmail.com (A.V.M.); 3 Frumkin Institute of Physical Chemistry and Electrochemistry, Russian Academy of Sciences, 119071 Moscow, Russia; marta.volovik@phystech.edu (M.V.V.); olegbati@gmail.com (O.V.B.); 4 State Research Center for Applied Microbiology & Biotechnology, 142279 Obolensk, Russia; korobova@obolensk.org (O.V.K.); borzilov@obolensk.org (A.I.B.); 5 Branch of M.M. Shemyakin & Yu.A. Ovchinnikov Institute of Bioorganic Chemistry, Russian Academy of Sciences, 142290 Pushchino, Russia; slascheva@bibch.ru (G.A.S.); dyachenko@bibch.ru (I.A.D.); 6 Department of Biotechnology, I.M. Sechenov First Moscow State Medical University, 119991 Moscow, Russia

**Keywords:** antibiotic, antimicrobial peptide, Gram-negative bacteria, lipopolysaccharide, polymyxin, drug resistance, in vivo studies

## Abstract

The rise in antimicrobial resistance represents a significant challenge to global health. The reason partially lies in an inappropriate use of conventional antibiotics and the subsequent rapid spread of multidrug-resistant pathogen strains. This emergency requires an urgent search for conceptually new antimicrobial agents. A viable alternative to conventional antibiotics is antimicrobial peptides (AMPs), which are ribosomally synthesized molecules with considerable potential as next-generation anti-infectious therapeutics. Previously, we have reported on the β-hairpin peptide Ap9, an analog of abarenicin from the marine polychaeta *Abarenicola pacifica*, with potent activity against key Gram-negative pathogens. Here, it is shown that Ap9 acts in a manner resembling polymyxin B, namely via interaction with lipopolysaccharide (LPS), and retains its activity against polymyxin-resistant isolates without observed cross-resistance, and causes insignificant damage in cytoplasmic membrane at bactericidal concentrations. NMR spectroscopy reveals that LPS binding induces a conformational rearrangement of Ap9, its dimer formation, and local structural remodeling of the peptide region (residues 8–12) into 3_10_-helix. Bacterial resistance to Ap9 was found to be relatively low with a reduced susceptibility associated with infrequent genetic alterations, such as the mutation in *lptD* or the deletion in *mlaA*. Furthermore, Ap9 demonstrates a favorable tolerability, a wider therapeutic window than that of polymyxin B, and a sufficiently long half-life through the systemic use, as well as in vivo efficacy in murine models of Gram-negative infections, including sepsis caused by the *mcr-1*-harboring *Escherichia coli* strain. The obtained results point to Ap9 as a promising candidate for further preclinical studies aimed at development of an alternative to polymyxins.

## 1. Introduction

According to the WHO 2024 bacterial priority list, *Escherichia coli*, along with *Acinetobacter baumannii*, *Klebsiella pneumoniae*, and *Pseudomonas aeruginosa* (key Gram-negative representatives of the ESKAPEE group) [1] are listed among pathogens of critical clinical concern [2]. Polymyxins (polymyxin B and colistin) remain one of the few classes of antibiotics effective against multidrug-resistant Gram-negative bacteria. Because of their pronounced nephrotoxicity and neurotoxicity, they are reserved as last-resort agents in the therapy of life-threatening infections. Although polymyxins exhibit pronounced antibacterial activities, several pathogen species, including *Proteus mirabilis*, *Neisseria meningitidis*, *Burkholderia* spp., and *Serratia* spp., display intrinsic resistance mediated by the constitutive expression of transferases such as EptA (PmrC), EptC, and ArnT. These enzymes catalyze the addition of phosphoethanolamine (pEtN) or 4-amino-deoxy-L-arabinose (L-Ara4N) to phosphate groups of lipid A, a key component of lipopolysaccharides (LPS), which is the primary target of polymyxins. These modifications reduce the overall negative charge of LPS, weakening its electrostatic interaction with cationic polymyxins. Additionally, certain bacteria have evolved defense strategies involving similar lipid A alterations controlled by two-component systems (PhoP/PhoQ and PmrA/PmrB) and regulatory elements (*arnBCADTEF-pmrE*, *pmrCAB*, *crrAB*, and *mgrB*). Over the past decade, plasmid-borne *mcr* genes (*mcr-1*–*mcr-10*), encoding pEtN transferases that modify lipid A at the 1′ or 4′ positions, have been identified in various species of Gram-negative bacteria [3]. The horizontal transfer of these genes has markedly accelerated the global spread of polymyxin resistance. In the bacterium *A. baumannii*, resistance to polymyxins can emerge as a consequence of spontaneous mutations in *lpxA*, *lpxC*, and *lpxD*, which impair lipid A biosynthesis and lead to LPS loss. Slight decreases in susceptibility to polymyxins may be caused by an increased production of anionic capsular polysaccharides (e.g., in *K. pneumoniae*), activation of efflux pumps (such as MtrCDE in *N. meningitidis* and AcrAB and KpnEF in *K. pneumoniae*), or overexpression of the outer membrane protein OprH in *P. aeruginosa* [4,5,6]. Consequently, these data emphasize the global dissemination of polymyxin resistance among Gram-negative bacteria and underscore the urgent need to develop novel antibacterial agents that exhibit enhanced pathogen selectivity and improved tolerability.

In our earlier study focused on the antibacterial potential of AMPs predicted from marine polychaete transcriptomes, we have identified the β-hairpin peptide abarenicin from *Abarenicola pacifica*, structurally related to arenicins from *Arenicola marina* [7]. Among arenicin-derived peptides with demonstrated in vivo efficacy, AA139 is one of the most prominent examples. The peptide has been obtained via structure-based optimization of arenicin-3, where structural data guided amino acid substitutions to balance antibacterial activity and toxicity [8]. Another arenicin-3 analog, NZ17074 (N1), displayed a strong antibacterial activity along with a significant toxicity, while its derivative N6, containing a single disulfide bond, showed a reduced toxicity and efficacy in murine models of *E. coli* peritonitis and LPS-induced endotoxemia [9]. Abarenicin itself exhibited a potent activity against Gram-negative bacteria but also displayed a marked cytotoxicity towards eukaryotic cells [7]. We performed alanine/arginine scanning in the β-turn region of abarenicin, a strategy we have previously applied to other β-hairpin peptides [10,11], resulting in a safer analog with an enhanced antibacterial activity, Ap9 (Y8A, M9R). The sequences and key physicochemical parameters of arenicin-like peptides are summarized in Table 1.

Ap9 demonstrated stability in serum, an antibiofilm activity against *P. aeruginosa*, and efficacy in murine models of *E. coli* infection. At concentrations far above the MIC (32 × MIC), Ap9 disrupted the bacterial cytoplasmic membrane, although its effects at lower concentrations were not assessed. Given its high activity against ESKAPEE strains, which in some cases exceeds that of polymyxin B, further research into this peptide as a prototype antibiotic is needed to better understand its properties. The present work aims to elucidate the mode of action of Ap9 in detail and to expand current knowledge of its antibacterial potential and safety profile.

## 2. Results

### 2.1. Ap9 Exerts Membranotropic Activity Without Inducing Stable Pore Formation or Complete Membrane Disruption at Concentrations Exceeding the MIC

**Figure 1:**
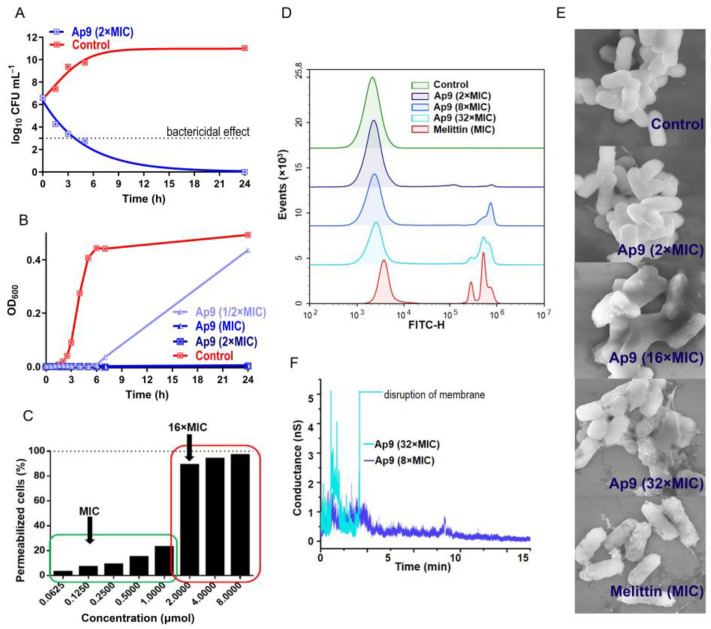
Investigation of membranotropic activity of Ap9. (**A**) Time-kill curves of Ap9 at 2 × MIC (MIC = 0.125 μmol) against *E. coli* ML-35p based on CFU counts. Data represent results from two independent experiments. (**B**) Growth inhibition kinetics of *E. coli* ML-35p measured by optical density at 600 nm, starting inoculum 10^6^ CFU mL^−1^. (**C**) Disruption of the cytoplasmic membrane integrity of *E. coli* ML-35p after 3 h incubation with Ap9 (ONPG assay). Data were normalized to melittin (8 μmol), which was set to 100% permeabilization. The green and red boxes indicate low and high levels of permeabilization following Ap9 treatment, respectively. The experiment was performed in three independent replicates. (**D**) Flow cytometry analysis of membrane permeabilization of *E. coli* ML-35p following 3 h exposure to Ap9, assessed by SYTOX Green uptake. Two independent experiments were performed, and similar results were obtained. (**E**) SEM images of *E. coli* ML-35p cells after 3 h treatment with Ap9 or melittin. (**F**) Kinetics of BLM conductivity induced by Ap9 at concentrations of 1 and 4 μmol. BLM lipid composition: PC (phosphatidylcholine), PE (phosphatidylethanolamine), PG (phosphatidylglycerol), cardiolipin (CL) at a molar ratio of 35:35:20:10.

To investigate the mechanism of Ap9 action in depth, we employed the *E. coli* ML-35p strain as the model in all assays, starting with the time-kill test to assess its bactericidal kinetics. As shown in Figure 1A, Ap9 exhibited a reasonably rapid, concentration-dependent bactericidal activity, achieving >3-log_10_ reduction in CFU within 3 h at 2 × MIC (MIC = 0.125 μmol), meeting the CLSI M26-A guideline for bactericidal activity [12]. Bacterial growth inhibition kinetics based upon optical density (OD_600_) measurements were consistent with CFU calculation results (Figure 1B).

Then, we examined whether Ap9 acts via a membranolytic mechanism, as is common for the majority of cationic AMPs. For this purpose, we performed a series of complementary assays. First, using ONPG and SYTOX Green, we evaluated the integrity of *E. coli* cytoplasmic membrane in the presence of Ap9 (Figure 1C,D). No significant permeabilization was detected at concentrations up to 8 × MIC in both assays, whereas loss of membrane integrity occurred only at higher concentrations. Scanning electron microscopy (SEM) confirmed the absence of detectable morphological changes on the surface of *E. coli* cells at 2 × MIC (Figure 1E). A pronounced surface deformation, including an increased roughness, dents, and leakage of cellular contents, was evident at ≥16 × MIC. In contrast, the membrane-active peptide melittin caused a comparable damage already at its MIC, while the arenicin-3 analog N6 disrupted the cytoplasmic membrane of *E. coli* within 5 min at 1 × MIC (PI-staining) [9]. In contrast, Ap9 demonstrated a concentration-dependent membranotropic activity, switching to a membrane destruction mechanism only at concentrations an order of magnitude higher than its MIC. Finally, the impact of Ap9 on bilayer lipid membranes (BLM), mimicking the negatively charged membrane of *E. coli*, was investigated (Figure 1F). The same types of conductance signals, including spike-like, stepwise, and multilevel, were detected at both 8 × MIC and 32 × MIC. According to [13,14], spike-like signals reflect the formation of lipid pores due to an imbalance of lateral pressure and tension between the contact and distal monolayers during the peptide adsorption. These pores then can expand by incorporating the peptide, resulting in toroidal pores (observed as stepwise signals) and peptide–lipid pores with fluctuating peptides at the edges (multilevel signals). At a lower concentration of Ap9 (8 × MIC), these pores were transient and tended to close spontaneously, whereas at a higher concentration (32 × MIC) their frequency and stability increased, ultimately leading to membrane rupture.

### 2.2. Resistance to Ap9 Is Linked to Specific Genetic Alterations

**Figure 2:**
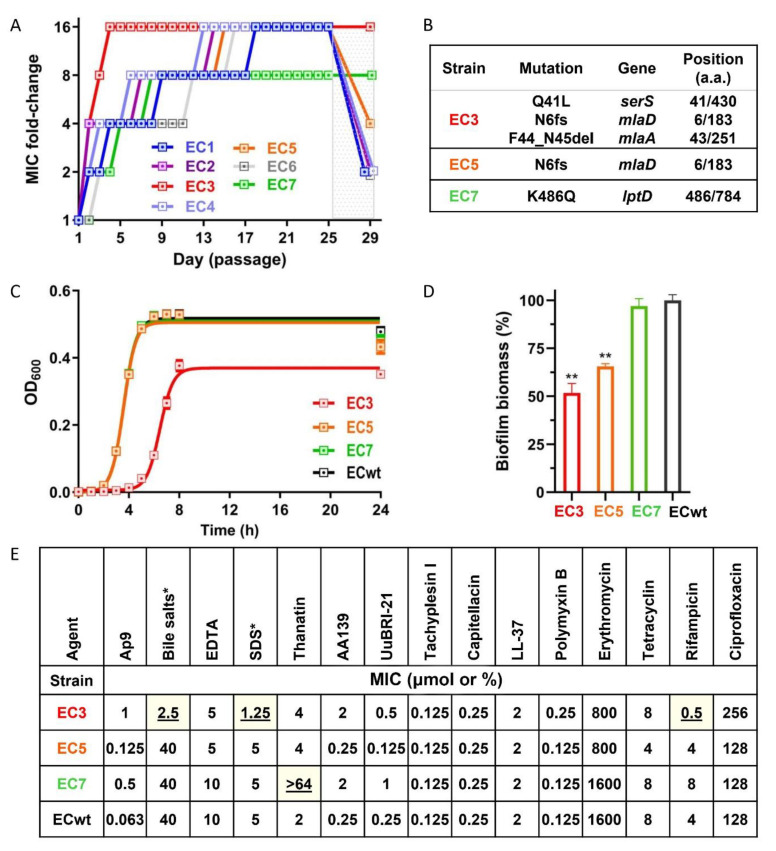
Analysis of resistance development to Ap9 in *E. coli* MDR CI 1057. (**A**) Induction of the *E. coli* MDR CI 1057 resistance development to the peptide. The initial MIC values in MH medium supplemented with 0.9% NaCl were 0.125 μmol. (**B**) Mutations associated with a decreased sensitivity to Ap9 in the isolated resistant strains. (**C**) Comparative growth rates of resistant strains. The experiment was performed in duplicate in two independent repeats. (**D**) Biofilm biomass formed by resistant strains, assessed by 0.1% crystal violet staining. (* *p* ≤ 0.05, ** *p* ≤ 0.01, in cases of a significant difference. (**E**) Cross-resistance profiling of resistant strains to various AMPs, antibiotics, and detergents. Values of ≥4-fold increases in the MIC, as compared to ECwt, are marked by pale yellow highlining and underlining.

The selection of antibiotic-resistant strains followed by whole-genome sequencing (WGS) facilitates elucidation of both key targets of the peptide and mechanisms of pathogen protection. To identify genes contributing resistance development to Ap9 in *E. coli* MDR CI 1057, we carried out selection experiments (Figure 2A). This strain carried two well-characterized mutations in the *gyrA* gene (S83L and D87N), which caused fluoroquinolone resistance and a higher-than-normal spontaneous mutation rate [15]. In four of seven cases, Ap9 induced a 16-fold increase in MIC over a 25-day period, which reverted to a two-fold increase following the drug-free passages, resulting in the final MIC of 0.25 µmol. Three other *E. coli* mutants with a decreased sensitivity to Ap9 (the final MIC ≥ 0.5 µmol) were isolated and subsequently analyzed using genome sequencing: EC3 (a 16-fold increase in the Ap9 MIC), EC5 (a four-fold increase), and EC7 (an eight-fold increase) (Figure 2B).

EC3 harbored mutations in the *serS* (missense), *mlaD* (frameshift), and *mlaA* (in-frame deletion) genes and exhibited a reduced growth rate and biofilm formation, along with an increased sensitivity to detergents, bile salts, and rifampicin, which together pointed to an enhanced outer membrane permeability (Figure 2C–E). A substantial increase in levels of inactivation of the Mla operon genes has been previously demonstrated under *E. coli* cells’ incubation with arenicin-3 [8]. By comparison, based upon an evolutionary screen across 14 unrelated AMPs, mutations in Mla components have been detected rarely, mainly under selection with pexiganan, HBD3, CAP18, or polymyxin B, without conferring a broad cross-resistance [16]. To date, a link between the *serS* gene and the development of resistance to antimicrobial agents has not been reported so far. It is known that deletions in the N-terminal arm-like domain (35–97 amino acid residues) reduce aminoacylation activity by approximately 10^3^-fold, whereas the single substitution of the conserved catalytic residue (E355Q) does not alter the enzymatic activity [17]. As the *serS* gene encodes seryl-tRNA synthetase, the enzyme involved in tRNA aminoacylation rather than in membrane biogenesis, it seems unlikely that the identified missense mutation (Q41L), located in the N-terminus outside the catalytic motif, plays a key role in the resistance development to Ap9. Conversely, mutations in the components of the Mla system, known to mediate the retrograde transport of mislocalized phospholipids from the outer leaflet of the outer membrane to the inner membrane, have represented a more plausible explanation for the altered membrane barrier function identified in EC3 [18]. The same *mlaD* mutation was observed in EC5, but this isolate showed no detectable morphological changes. This indicates that mutations in the *mlaA* gene resulting in deletion of the residues F44 and N45 in the N-terminal region of the protein likely makes a major contribution to the EC3 phenotype. Previously, Sutterlin et al. have described a dominant gain-of-function allele of the *E. coli mlaA*, termed mlaA*, characterized by the two-residue deletion (ΔN41-F42) which led to the accumulation of phospholipids in the outer leaflet of the outer membrane, even in the absence of other Mla components [19]. Notably, both EC3 (ΔF44-N45) and mlaA* (ΔN41-F42) resulted in the same mutant MlaA structure. The mlaA* allele disrupted lipid homeostasis and activated *pldA*, which encodes phospholipase A, resulting in increased levels of LPS in particular hepta-acylated forms, the depletion of inner membrane phospholipids, and consequent outer membrane vesiculation. Although the authors have reported that additional mutations in other components of the Mla system (e.g., Δ*mlaF* or Δ*mlaD*) on the mlaA* background did not weaken the observed phenotype, a closer look at plating efficacy assays suggested that these double mutants appeared to form slightly more colonies than the mlaA* alone, supposing an activation of compensatory mechanisms that might partially restore the outer membrane integrity. In our case, a similar deletion in *mlaA* together with Δ*mlaD* resulted in the viable strain EC3, characterized by an increased outer membrane permeability, in contrast to the lethal phenotype associated only with mlaA*.

Abellor-Ruiz et al. have determined the crystal structure of *K. pneumoniae* MlaA and have suggested that the corresponding two-residue deletion (ΔN43-F44) might destabilize the helix H1 and/or its interaction with the pore loop, creating a breach in the channel wall that could allow inner leaflet phospholipids to enter the pore [20]. Taken together, we propose that the identified deletion in *mlaA* results in a significant remodeling of inner and outer cell membranes, accompanied by the formation of LPS-containing vesicles, which may ultimately reduce the effectiveness of the peptide interaction with the target cell. Earlier, it has been reported that after 18 serial passages in the presence of the arenicin-3 analog N6, *E. coli* mutants resistant to the peptide were not found [9]. In contrast, it has been shown that in *E. coli* clones resistant to N6, the *mlaA* gene was likewise repeatedly mutated (L10Q, V213fs, or N45_V46_ins_FN) along with alterations in other genes of outer membrane proteins, such as BamA and envelope-associated ones as well as some transcriptional regulators [21]. It is noteworthy that the presence of these mutations results in a markedly reduced virulence, suggesting that such adaptations impose significant fitness costs [21]. The reduced sensitivity to Ap9 may also be associated with a mutation in another outer membrane β-barrel protein LptD, an essential protein of the Lpt complex (LptA–LptG) that is responsible for LPS transport across the bacterial envelope. The final step of LPS insertion into the outer leaflet of the outer membrane is mediated by the LptD/LptE translocon [22,23].

Further, we set a goal to find out whether the resistance to Ap9 conferred a cross-resistance to other AMPs or conventional antibiotics (Figure 2E). EC3 exhibited a reduced sensitivity to AA139 (an eight-fold increase in the MIC), while EC7 was resistant to AA139 (an eight-fold increase) and UuBRI-21 (a four-fold increase), as well as to thanatin (>a 32-fold increase). The last-mentioned peptide has been previously reported to interact with LptA and LptD [24]. Notably, none of the strains displayed the cross-resistance to polymyxin B, which targets the outer bacterial membrane. Vice versa, no cross-resistance effects were observed for bacteria exhibiting the intrinsic or acquired resistance to polymyxin B: the *E. coli* strain bearing the plasmid containing the *mcr-1* gene, the polymyxin-resistant *E. coli* strain P1 (a 128-fold MIC increase; harboring mutations in *pmrB*) [25] and *P. mirabilis* (Supplementary Table S2). The cross-resistance to AA139 and UuBRI-21 is consistent with their high structural similarity to Ap9, whereas the retained susceptibility to other β-hairpin AMPs, capitellacin and tachyplesin I, indicates rather different mechanisms of action [26].

### 2.3. Ap9 Likely Interacts with LPS

Although the identified LptD mutation (K486Q) appears to be sterically unfavorable for direct interaction with Ap9 (Supplementary Figure S3), we nevertheless assessed binding of the peptide with LptD as its potential target using the microscale thermophoresis (MST) method for quantitative analysis of protein–ligand interactions under conditions similar to physiological ones [27,28]. Furthermore, an eight-fold increase in the MIC for EC7 (up to 1 μmol) suggests a shift in the mechanism of action to the membrane disruption (Figure 1), if peptide binding to the target protein on the cell surface becomes impossible due to the mutation. As even a minor modification can markedly influence structures and activities of relatively short AMPs [29,30], we opted to attach a fluorescent tag via a flexible GSGS linker to the lipoprotein LptE, which is required for proper folding of LptD into the native “barrel and plug” architecture. Accordingly, LptD and LptE were co-expressed in *E. coli* BL21(DE3) together with the green fluorescent protein (GFP) and a hexahistidine tag fused to the C-terminus of LptE. Western blot analysis, SDS-PAGE, and mass spectrometry confirmed the presence of the recombinant LptD and LptE-GFP-His_6_, and their molecular masses were proved to correspond to the calculated ones of 89,653.2188 Da and 49,292.1015 Da, respectively (Supplementary Figure S4A,B). GFP labeling appeared to enhance stability of the complex, as indicated by the higher expression level of LptD/LptE-GFP-His_6_ when compared to the unlabeled complex (Supplementary Figure S4C).

**Figure 3:**
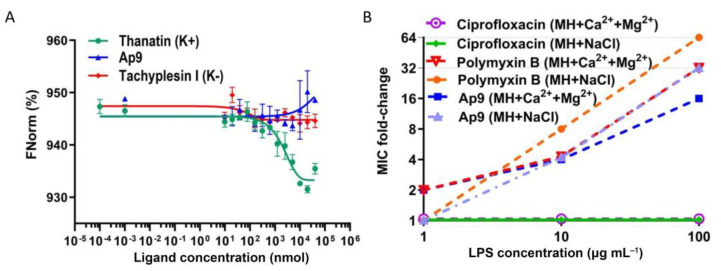
Analysis of Ap9 interaction with the LptD/LptE-GFP-His_6_ complex and influence of LPS on the peptide activity. (**A**) MST dose–response curves showing binding of thanatin, Ap9, and tachyplesin I to the LptD/LptE-GFP-His_6_ complex. Peptide (ligand) concentrations ranged from 1 nmol to 40 μmol, while the complex concentration was constant (300 nmol). Measurements were performed at 20% MST and 100% LED powers. (**B**) Antimicrobial activities of Ap9, polymyxin B, and ciprofloxacin against *E. coli* ML-35p in the presence of LPS. Initial MICs: 0.125 μmol for Ap9 and polymyxin B, 0.063 μmol for ciprofloxacin.

Expectedly, MST experiments demonstrated no interaction of Ap9 with the LptD/LptE-GFP-His_6_ complex, even at concentrations well above its MIC (Figure 3A). In contrast, the reference peptide thanatin (the MIC value of 2 μmol against *E. coli*) bound to the complex with an apparent affinity in the micromolar range (*K*_d_ = 1.893 ± 0.753 μmol). The deviation from previously reported *K*_d_ for thanatin can be explained by methodological differences [24], particularly in labeling the peptide instead of the protein complex.

Notably, the resistance to the antibiotic murepavadine (POL7080) targeting LptD in *P. aeruginosa*, is predominantly associated with mutations in the *pmrB* gene that modify LPS, thereby conferring the cross-resistance to colistin [31]. These data, together with elucidation of the central role of the LptD/LptE complex in LPS transport, highlight the close link between LptD and LPS in mediating resistance development to antibiotics targeting the outer membrane and its protein components. We supposed that the identified mutation in LptD may affect the Ap9 antimicrobial activity indirectly through the altered LPS transport, while Ap9 likely interacts directly with LPS.

Then, we evaluated the impact of LPS on Ap9 activity against *E. coli* ML-35p under two different conditions: in the medium supplemented with 0.9% NaCl and in the medium containing divalent cations, which strengthen lateral interactions between LPS molecules and enhance outer membrane barrier properties (Figure 3B) [32]. Polymyxin B, binding LPS [33], was used as a positive control, and ciprofloxacin, a DNA gyrase/topoisomerase inhibitor lacking LPS affinity [34], served as a negative control. The addition of LPS significantly reduced the Ap9 activity in a similar way to that observed for polymyxin B, but had no effect on the ciprofloxacin action. However, the reduction was less pronounced for Ap9 (a 16–32-fold increase in the MIC depending on conditions) than for polymyxin B (a 32–64-fold increase). This finding suggests that the Ap9 selectivity is likely associated with its binding to LPS, although its antibacterial activity is not exclusively contingent on this interaction.

### 2.4. Ap9 Undergoes Dimerization and Conformational Change in the Presence of LPS

**Figure 4:**
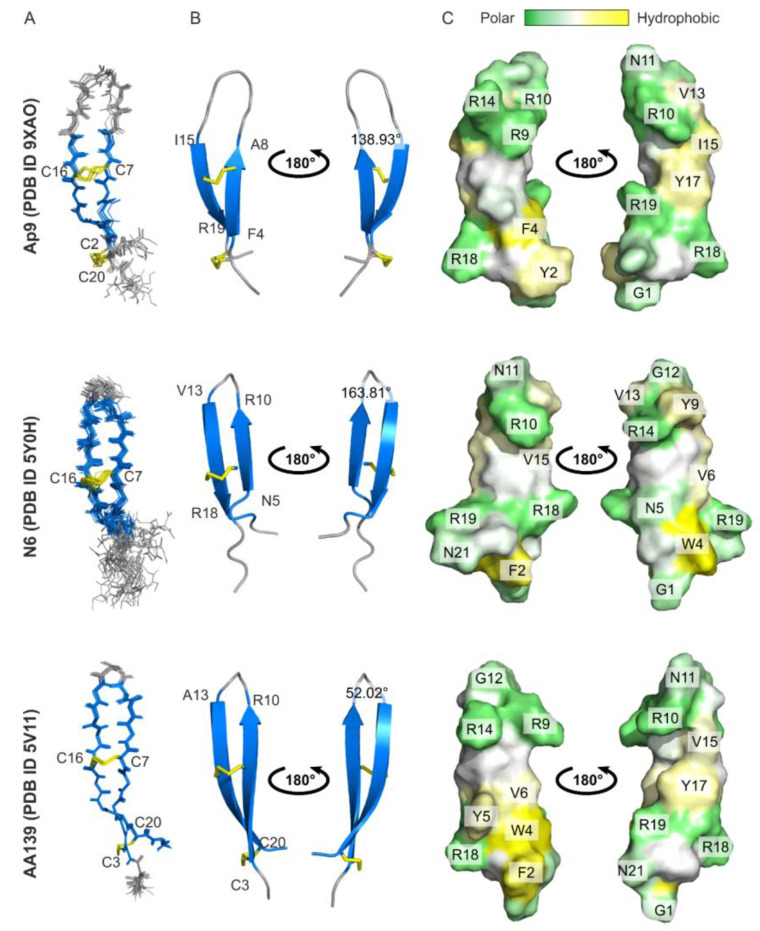
Spatial structures of Ap9 and arenicin-3-derived AMPs (AA139, N6) in aqueous solution. (**A**) Set of 10 NMR structures of the peptides with the fewest restraint violations. Disulfide bonds are highlighted in yellow. (**B**) Ribbon representation of spatial structures of the peptides with loop regions shown in gray and β-sheets in blue. Disulfide bonds are highlighted in yellow. (**C**) Distribution of hydrophobic potential on the surface of the molecules. Polar regions are shown in green, hydrophobic regions in yellow. All data are provided for Ap9 (PDB ID: 9XAO), N6 (PDB ID: 5Y0H), AA139 (PDB ID: 5V11).

The arenicin-3 analog N6 has been previously shown to bind LPS with a high affinity in the nanomolar range as determined by isothermal titration calorimetry (ITC) method [21]. However, no structural data describing the peptide conformational changes upon LPS binding are available. To address this knowledge gap, we first determined the solution structure of Ap9 in water and then compared it with two closely related arenicin-3 analogs AA139 (PDB ID: 5V11) and N6 (PDB ID: 5Y0H) (Figure 4).

In aqueous solution, a total of 100 structures of Ap9 were calculated using the torsion angle, upper NOE-based distance, hydrogen bond, and disulfide bond restraints (Supplementary Table S3). The final set of 10 NMR structures demonstrated low RMSD values for backbone atoms (0.36 ± 0.16 Å) and heavy atoms (0.97 ± 0.20 Å) with no significant restraint violations, indicating that the peptide structure is well defined (Figure 4A, Supplementary Figures S5A and S6A). Ap9 adopts an antiparallel β-sheet (F4–A8 and I15–R19) connected by a loop region (Figure 4A). The conformation is stabilized by two disulfide bridges (C3–C20 and C7–C15) and five hydrogen bonds.

A comparison of Ap9 with AA139 and N6 revealed several structural differences. The size of β-sheet varies among the peptides: in Ap9, the strands involve amino acid residues 4–8 and 15–19; in N6, they include residues 5–10 and 13–18, whereas in AA139, nearly the entire sequence contributes to the formation of a β-hairpin (Figure 4A,B). The β-hairpin turn angle demonstrates a substantial variation, exhibiting the greatest magnitude in N6 (163.81°), the most intermediate value in Ap9 (138.93°), and the least in AA139 (52.02°) (Figure 4B). A distinctive feature of Ap9 is the presence of two hydrogen bonds between residue pairs A8–I15 and A6–Y17, which are absent in AA139 and N6. Despite these structural variations, the overall distribution of surface charge and hydrophobic potential is broadly similar across the three peptides (Figure 4C).

**Figure 5:**
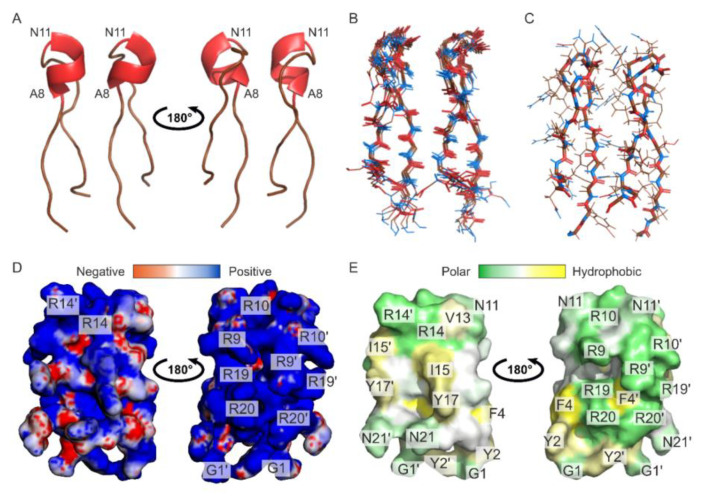
Three-dimensional structure of the dimeric Ap9. (**A**) The ribbon representation of Ap9. Red color shows the regions of 3_10_-helix, brown color indicates the loop regions of the peptide. (**B**) Set of 10 NMR structures of Ap9 with the fewest restraint violations. Data on the main chain of the peptide are presented. NH and CO groups are colored in blue and red, respectively. (**C**) The peptide structure with side groups is shown. (**D**) Electrostatic surface potential of the dimeric Ap9 in free solution. The data were obtained using the APBS tools. (**E**) Distribution of hydrophobic potential on the molecular surface. The polar regions are shown in green, and hydrophobic regions in yellow. Residues from chain A are denoted without additional markers, while the chain B residues are identified using the ‘ symbol.

The addition of LPS at a peptide-to-lipid ratio of 2:1 induced the aggregation of Ap9, as indicated by the precipitate formation and the disappearance of signals in the ^1^H-, ^1^H,^15^N-, ^1^H,^13^C-HSQC, ^1^H,^1^H-TOCSY, ^1^H,^1^H-NOESY spectra. Increasing the peptide-to-lipid ratio to 10:1, as previously applied for another LPS-binding AMP thanatin [35], maintained the peptide solubility and revealed new spin systems in the ^1^H,^1^H-TOCSY spectra (Supplementary Figure S7A). The correlation of these signals with the ^1^H,^1^H-NOESY spectra enabled the assignment of A8, R9, N11, G12, and Y2 residues. The detection of new cross-peaks consistent with an altered peptide state, together with distinct intermolecular NOE cross-peaks, suggested a dimer formation with intermolecular contacts between amino acid residues 9, 10, 13–20 of chain A and residues 2–8 and 12 of chain B (Supplementary Figure S7B, Figure 5). The dimeric peptide structure was calculated using analogous restraints as was for the monomeric form of Ap9. The final set of NMR structures demonstrated a well-defined structure with low RMSD values for backbone atoms (0.47 ± 0.14 Å) and heavy atoms (1.33 ± 0.23 Å) (Supplementary Table S3, Figure 5, Supplementary Figure S5B). In the dimer, Ap9 contains 3_10_-helix (A8–N11) flanked by two unconstrained regions (G1–A8 and G12–N21), and the dimer is stabilized by four disulfide bridges (C3–C20 and C7–C15 per monomer) and 16 hydrogen bonds (Figure 5A,B,D). Electrostatic analysis indicates that the molecular surface of the dimer is polar with a strong positive charge contributed by arginine residues from both chains on one side and a neutral region formed by residues 15–21 on the other side (Figure 5C). Compared with the monomer, residues 8–12 undergo local conformational remodeling and adopt 3_10_-helix instead of a loop (Figure S7C). This rearrangement redistributes the electrostatic potential, forming a neutral area (residues 15–21 of both chains), which may facilitate interactions with the membrane environment. These observations are consistent with a role for LPS in Ap9 activity against Gram-negative pathogens and prompted us to further evaluate its therapeutic potential in vivo. However, the quantitative characterization of the peptide binding to LPS remains to be established.

### 2.5. Ap9 Is Well-Tolerated and Efficacious in Murine Models of P. aeruginosa and Polymyxin-Resistant E. coli Infections and Has a Favorable In Vivo Stability

**Figure 6:**
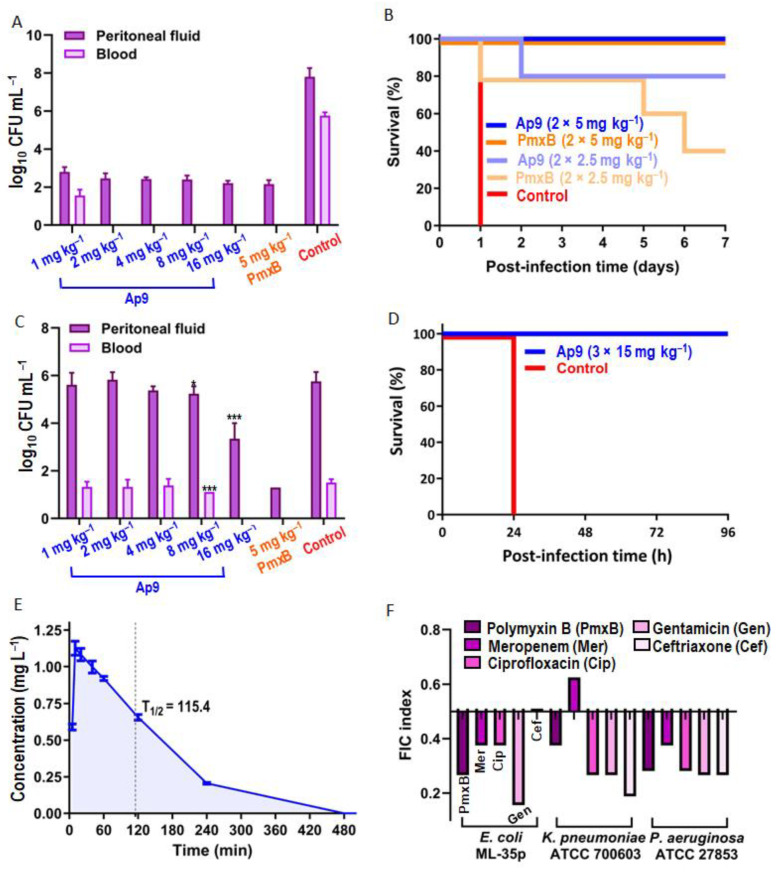
Therapeutic efficacy and pharmacokinetics of Ap9 in animal models. (**A**) Minimal therapeutic dose of Ap9 in peritonitis model in cyclophosphamide-treated BALB/c mice (*n* = 5), 6 h after intraperitoneal infection with virulent *E. coli* 3421E/19 (1 × 10^6^ CFU per animal). (**B**) Survival of BALB/c mice (*n* = 5) infected with uropathogenic *E. coli* U10 (1 × 10^7^ CFU per animal in the presence of 2.5% mucin) after treatment with Ap9. (**C**) Minimal effective dose of Ap9 in peritonitis models in BALB/c mice (*n* = 5), 6 h after intraperitoneal infection with *P. aeruginosa* PAO1 (10^5^ CFU per animal in the presence of 2.5% mucin). * *p* < 0.05; *** *p* < 0.001. (**D**) Survival of BALB/c mice (*n* = 5) infected with *P. aeruginosa* PAO1 (1.6 × 10^5^ CFU per animal in the presence of 2.5% mucin) after Ap9 treatment. (**E**) Plasma concentration–time profile of Ap9 in SD rats (*n* = 3) after a single intramuscular injection (1 mg kg^−1^), measured by LC-MS/MS. The half-life of Ap9 (T_1/2_) is indicated by the dotted line. (**F**) FIC indices of antibiotics in combination with Ap9 by action against key Gram-negative bacteria, determined using the checkerboard assay. FIC values of ≤0.5 indicate synergy. Data represent results from three independent experiments.

Previously, we have used AA139 as a reference peptide and have shown that, in the murine sepsis model with *E. coli* ATCC 25922, both Ap9 and AA139 at the same doses displayed strong in vivo activities with survival rates of 100% and 87.5%, respectively [7]. Moreover, a single administration of Ap9 (10 mg kg^−1^) markedly reduced CFU in the blood and peritoneal fluid of BALB/c mice infected with the virulent isolate *E. coli* 3421E/19. Here, we demonstrated that Ap9 was effective even at a dose of 1 mg kg^−1^ in the same murine model, indicating a higher antibacterial potency in vivo (Figure 6A). To further explore this, and considering the absence of cross-resistance with polymyxin B, we assessed the peptide efficacy in the murine sepsis model caused by the *E. coli* strain U10 harboring the *mcr-1* gene. Antimicrobials at the doses listed below were used twice at 4 h intervals. Treatment with Ap9 of mice infected with U10 resulted in complete protection at a dose of 5 mg kg^−1^ and 80% survival at 2.5 mg kg^−1^ (Figure 6B). Polymyxin B also provided 100% survival at a dose of 5 mg kg^−1^, whereas at 2.5 mg kg^−1^, survival dropped to 40%. Although polymyxin B showed an efficacy at a dose of 5 mg kg^−1^ (cumulative 10 mg kg^−1^), its maximum tolerated dose (MTD) in mice has been defined as 5.4 mg kg^−1^ after a single intravenous injection (i.v.) [36]. In contrast, Ap9 was safe in ICR mice up to a dose of 15 mg kg^−1^ (single i.v.), without detectable changes in behavior, relative organ weights, or histopathological alterations after either 24 h or 14 days (Supplementary Table S4). At the same time, AA139 at a single 15 mg kg^−1^ i.v. dose induced mild-to-moderate histological changes in the liver and kidneys. Neurotoxicity and organ pathology were observed at doses ≥ 30 mg kg^−1^ for both peptides. These data demonstrate that Ap9 retains its activity at the above-mentioned doses where polymyxin B cannot be utilized due to its ineffectiveness or the associated risk of adverse effects. This provides a broader therapeutic window for the treatment of bacterial infections with diminished sensitivities to polymyxins.

After confirming Ap9 efficacy in several murine models of *E. coli* infection, we focused on another clinically important pathogen of the ESKAPEE group. Since Ap9 demonstrated potent in vitro antibacterial and antibiofilm activities against diverse *P. aeruginosa* strains [7], we evaluated its minimal effective dose in vivo against the reference strain PAO1 (Figure 6C). A pronounced reduction in bacterial burden was achieved only at the highest tested dose (16 mg kg^−1^). In view of data on the safety profile of Ap9, its efficacy was assessed in the sepsis murine model with *P. aeruginosa* PAO1, using the dose of 15 mg kg^−1^. Three i.p. injections of the peptide conferred 100% survival (Figure 6D) without observable signs of toxicity. In this experiment, polymyxin B at the same i.p. dose was lethal to all animals within 24 h.

Finally, a pharmacokinetic study of Ap9 in SD rats after a single intramuscular administration at a dose of 1 mg kg^−1^ showed a half-life of approximately 2 h (115.4 min) with an area under the curve (AUC) of 180.4 mg∙min L^−1^, suggesting its sufficient systemic stability (Figure 6E). For comparison, the half-life of polymyxin B in the plasma of rats has been reported as 1.45 h (87 min) after administration of 1 mg kg^−1^ i.v. [36].

### 2.6. Ap9 Exhibits a Pronounced Synergism with Conventional Antibiotics When Acting on ESKAPEE Bacteria

In addition to the individual antibacterial activity of the peptide, we evaluated effects of Ap9 in combination with antibiotics of different classes against model strains of clinically relevant Gram-negative species (Figure 6F). The peptide displayed synergistic effects with polymyxin B (polymyxins), meropenem (carbapenems), ciprofloxacin (fluoroquinolones), gentamicin (aminoglycosides), and ceftriaxone (cephalosporins) by action against *E. coli* ML-35p, *P. aeruginosa* ATCC 27853, and *K. pneumoniae* ATCC 700603. The only exception was the Ap9–meropenem combination, which showed an additive effect (FICI = 0.625) when acting against *K. pneumoniae*. Synergistic or additive effects may be explained by the ability of Ap9 to disrupt outer membrane integrity, thereby enabling antibiotics to reach their periplasmatic or cytoplasmic targets. For instance, meropenem and ceftriaxone inhibit penicillin-binding proteins (PBPs) [37], gentamicin targets the 30S ribosomal subunit, and ciprofloxacin blocks DNA gyrase and topoisomerase IV [38]. In the case of polymyxin B, which is also a membrane-active antibiotic, the observed synergy may be attributable to a complementary, albeit divergent, mode of action on bacterial cells and their membranes.

Being effective as an individual antimicrobial agent, Ap9 also seems to enhance activities of conventional antibiotics against multidrug-resistant Gram-negative pathogens. Moreover, given Ap9′s ability to bind LPS, its use as an anti-inflammatory agent in combination with bactericidal antibiotics may also be considered and addressed in more detail.

## 3. Discussion

**Figure 7:**
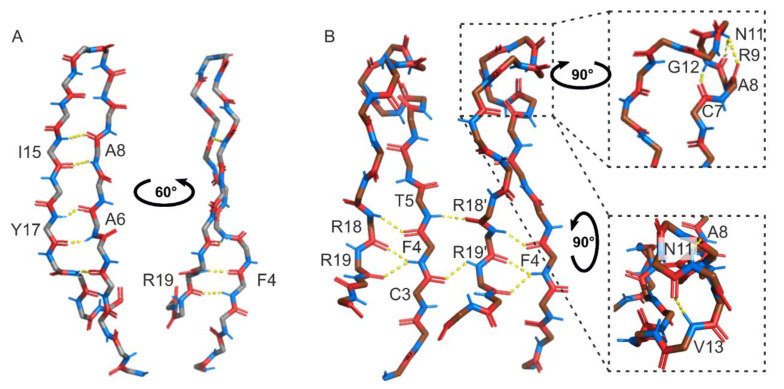
Positions of hydrogen bonds in the monomeric (**A**) and dimeric (**B**) forms of Ap9. Yellow dotted lines represent hydrogen bonds (≤2.4 Å). NH and CO groups are indicated in blue and red, respectively. Amino acid residues from chain A are denoted without additional markers, while the chain B residues are identified using the ‘ symbol.

In the present study, we investigated the mechanism of action of the abarenicin-derived peptide Ap9 and its anti-infective effects in vivo. NMR spectroscopy revealed that in the presence of LPS Ap9 underwent conformational remodeling and formed the dimer in which the peptide region containing amino acid residues 8–12 adopted 3_10_-helix instead of a loop observed in the monomer (Supplementary Figure S7C). In the dimer, new hydrogen bonds (V13–N11, G12–C7, N11–R9/A8) formed and stabilized the 3_10_-helical segment, whereas several hydrogen bonds (A8–I15, A6–Y17) in the β-sheet region disappeared, leading to an increased disorder in this area (Figure 7). The dimeric form of the peptide is further stabilized by two intermolecular hydrogen bonds (T5–R18′, C3–R19′). These LPS-mediated structural changes result in a redistribution of electrostatic potential, creating an amphipathic structure with a hydrophobic surface patch (residues 15–21 of both chains, Figure 5E) and an extensive positively charged area (Figure 5D), which could promote further selective interactions of Ap9 with membranes of Gram-negative bacteria. Moreover, comparison of Ap9 with AA139 and N6 indicates that Ap9 possesses a loop segment of a higher conformational lability, which may account for its propensity to undergo remodeling in the presence of LPS. To our knowledge, these data represent the first structural evidence of such a conformational rearrangement in β-hairpin AMPs upon LPS binding.

Unlike most typical membrane-active AMPs disrupting the cytoplasmic membrane integrity at or near their MICs [26,39,40,41], Ap9 damaged the outer membrane (Supplementary Figure S8) and exerted a bactericidal activity without complete cytoplasmic membrane permeabilization/disruption, up to 8 × MIC. BLM recordings at this concentration showed conductance events consistent with reversible, transient lipid and toroidal peptide–lipid pores rather than stable pore formation. We therefore suggest that the outer membrane perturbation is not sufficient to explain the effect of bacteria killing. Notably, Ap9 did not inhibit bacterial translation even at concentrations up to 128 × MIC, indicating that macromolecular synthesis does not account for its antibacterial activity (Supplementary Figure S9). Instead, bactericidal activity of the peptide is more likely mediated by downstream destabilization of transmembrane processes rather than by direct cytoplasmic membrane lysis (Supplementary Figure S10).

Next, we took aim to find out which pathways mediate adaptation to Ap9. Resistance to Ap9 was relatively low and associated with mutations in either the Lpt or the Mla pathways, responsible for LPS insertion and retrograde phospholipid transport across the outer membrane, respectively. Previous studies on arenicin-3 analogs have reported variable resistance outcomes without evidence for a defined molecular target [9,21,42]. Together with our results, these observations made it unlikely that proteins of Mla-complex are direct targets of arenicin-3 and, alternatively, indicated that resistance arises through a broader envelope defense strategy [8]. In general, bacteria adapt either by: (a) mutations in a specific target, or (b) reducing peptide uptake via efflux pump genes expression and remodeling of the cell envelope (surface charge, altered cell-wall/lipid metabolism, etc.). For Ap9, our data clearly support the latter route. The resistance-linked mutations were associated with an increased outer membrane vesiculation and changes in LPS abundance that could reduce the effective concentration of Ap9 on the target cell surface.

Ap9 also displayed a strong synergy with polymyxin B by action against several Gram-negative species and retained the activity against polymyxin-resistant *E. coli* isolates both in vitro and in vivo, supporting the idea that the peptide interacts with distinct sites in LPS. This lack of cross-resistance along with its high in vivo efficacy underscores the therapeutic potential of Ap9. At the same time, AA139 and N6 represent the few closely related arenicin-like β-hairpin AMPs under investigation, emphasizing the strategic importance of developing a pool of structurally similar molecules, since they may exhibit different activity–safety profiles and consequently improve the chances of preclinical success. A summary of the key properties of Ap9 and related arenicin-derived peptides is presented in Supplementary Table S5.

## 4. Materials and Methods

### 4.1. Recombinant Production of AMPs

AMPs, except LL-37 and melittin, were expressed in *E. coli* BL21 (DE3) as fusion proteins containingthe following elements: His-tag, thioredoxin A, methionine residue, and the mature peptide, as previously described [7]. For in vivo studies, Ap9 and AA139 were produced using the endotoxin-free ClearColi^®^ BL21(DE3) expression system. LL-37 and melittin (>98% purity) were obtained using standard solid-phase peptide synthesis.

### 4.2. Antibacterial Activity

Antibacterial activity was assessed by two-fold serial dilutions in sterile 96-well flat-bottom microplates using Mueller-Hinton broth supplemented with 0.9% NaCl (MH + NaCl). Bacterial cultures were grown in LB medium at 37 °C up to the optical density at 600 nm (OD_600_) of 1.0 and diluted in MH + NaCl to 1 × 10^6^ CFU mL^−1^. Test cultures (50 μL) were combined with equal volumes of peptide solutions prepared in 0.1% sterile bovine serum albumin (BSA) to minimize nonspecific adsorption to the plate surface. Plates were incubated at 37 °C for 24 h with shaking at 950 rpm. Minimum inhibitory concentrations (MICs) were defined as the lowest peptide concentrations that prevented bacterial growth, determined either visually or using the resazurin reduction assay. Values represent median MICs from three independent experiments performed in triplicate.

The influence of LPS on antibacterial activities of Ap9, polymyxin B, and ciprofloxacin against *E. coli* ML-35p was assessed using the same protocol as above, with the medium supplemented with LPS (*E. coli* O55:B5, ≥99%, ServiceBio, Wuhan, China) at final concentrations of 1, 10, and 100 μg mL^−1^.

### 4.3. Time-Kill Assay

An overnight culture of *E. coli* ML-35p was diluted in LB medium to OD_600_ of 1.0. Bacterial cells were mixed with Ap9 at concentrations equal to 2 × MIC (0.25 μmol), 8 × MIC (1 μmol) and 32 × MIC (4 μmol) in a 96-well microplate and incubated at 37 °C with shaking at 950 rpm. At 0, 1, 2, 3, 6, 8, and 24 h, aliquots of treated cells were withdrawn, serially diluted in 0.9% NaCl, and 10 μL of each dilution was spotted onto LB agar plates. Plates were incubated at 37 °C for 18 h, after which colony counts were performed in each dilution yielding from 50 to 250 colonies. The untreated culture served as a growth control. Bactericidal activities were defined as a 99.9% reduction in *E. coli* counts (a decrease of >3 log_10_ CFU mL^−1^), relative to the corresponding controls at each time point.

### 4.4. Growth Inhibition Kinetics

*E. coli* ML-35p growth was monitored by an optical density at 600 nm (OD_600_). An overnight culture was diluted in LB medium to OD_600_ of 1.0 and incubated with Ap9 at 0.5×, 1×, and 2 × MIC (0.0625, 0.125, and 0.25 μmol, respectively). OD_600_ was recorded at 0, 0.5, 1, 1.5, 2, 2.5, 3, 4, 5, 6, 7, and 24 h using a microplate reader AF2200 (Eppendorf, Hamburg, Germany). The untreated culture served as a growth control.

### 4.5. Outer Membrane Permeability Assay

*E. coli* ML-35p was grown overnight at 37 °C in 3% trypticase soy broth (TSB). Cells were harvested (4 °C, 5000 rpm, 10 min) and washed three times with cold phosphate-buffered saline (PBS, pH 7.4). The pellet was resuspended in PBS containing 0.9% NaCl to obtain the stock suspension containing 2.5 × 10^8^ CFU mL^−1^. 50 μL of each peptide solution were prepared in 0.1% BSA and combined in a 96-well flat-bottom microplate with 150 μL of the obtained bacterial suspension premixed with nitrocefin (Calbiochem, Darmstadt, Germany) to achieve a final concentration of 20 μmol. Nitrocefin hydrolysis was monitored at 492 nm using a microplate reader AF2200 (Eppendorf, Hamburg, Germany). The experiment was performed in three independent replicates, and curve patterns were similar in all cases.

### 4.6. Inner Membrane Permeability Assays

ONPG-test: *E. coli* ML-35p was grown overnight at 37 °C in 3% trypticase soy broth (TSB), and then cells were harvested (4 °C, 5000 rpm, 10 min) and washed three times with cold phosphate-buffered saline (PBS, pH 7.4). The obtained pellet was resuspended in PBS supplemented with o-nitrophenyl-β-D-galactopyranoside (ONPG, Darmstadt, AppliChem GmbH, Germany) and NaCl to obtain the stock suspension of 2.5 × 10^8^ CFU mL^−1^. 50 μL of peptide solutions were prepared in 0.1% BSA and were mixed with 150 μL of the bacterial suspension. The final reaction volume was 200 μL and contained 2.5 × 10^7^ CFU mL^−1^ cells, 2.5 mmol ONPG, and 0.9% NaCl. After 3 h, o-nitrophenol—the yellow chromogenic product of ONPG hydrolysis, indicating cytoplasmic membrane permeabilization—was monitored spectrophotometrically at 405 nm using microplate reader AF2200 (Eppendorf, Hamburg, Germany). The assay was performed in three independent replicates, and curve patterns were similar in all cases.

SYTOX Green uptake assay by flow cytometry: *E. coli* ML-35p cells were prepared identically to the ONPG assay and incubated with peptides for 3 h at 37 °C. Then, SYTOX Green (Life Technologies, Carlsbad, CA, USA) was added to treated cells to the final concentration of 2.5 μmol and incubated for 15 min in the dark. Samples were analyzed on a Novocyte 2060R flow cytometer (ACEA Biosciences Inc., San Diego, CA, USA) at 488 nm and 530 nm. Obtained data were processed using NovoExpress Software v. 1.2.4 (ACEA Biosciences Inc., San Diego, CA, USA). Two independent experiments were performed, and similar results were obtained.

### 4.7. Scanning Electron Microscopy (SEM)

*E. coli* ML-35p was grown to the mid-log phase (OD_600_ 0.7) at 37 °C, pelleted, washed three times with sterile PBS (pH 7.4), and adjusted to 1 × 10^6^ CFU mL^−1^. Aliquots were incubated with Ap9 at concentrations equal to 2 × MIC (0.25 μmol), 16 × MIC (2 μmol), and 32 × MIC (4 μmol) for 3 h at 37 °C. The obtained samples were placed on silicon wafers coated with a carbon film and air-dried at room temperature. SEM imaging was carried out on a MAIA3 electron microscope (Tescan, Brno, Czech Republic) at 7 kV accelerating voltage.

### 4.8. BLM Measurements

Planar bilayer lipid membranes (BLM) were formed by the Muller–Rudin method on the copper mesh rings affixed to the bottom of a Petri dish filled with the buffer solution (100 mmol KCl, 10 mmol HEPES, pH 7.4). BLM were formed from a lipid mixture composed of phosphatidylcholine (PC), phosphatidylethanolamine (PE), phosphatidylglycerol (PG), and cardiolipin (CL) at a molar ratio of 35:35:20:10. The ground electrode was placed in the buffer solution within the Petri dish, while the measuring electrode was connected to the micropipette filled with the same buffer containing Ap9 at concentrations equal to 8 × MIC (1 μmol) and 32 × MIC (4 μmol). The micropipette position was monitored using microcontrollers and a camera on the computer screen. After a tight contact between the micropipette and BLM, a constant voltage of +100 mV was applied to the membrane using a patch-clamp amplifier (HEKA EPC-8, HEKA Elektronik, Lambrecht, Germany), and the membrane conductance was recorded for 15 min.

### 4.9. Resistance Induction Assay

An overnight culture of *E. coli* MDR CI 1057 (SCPM-O-B-10910, GenBank accession: SRS24242713) was diluted in MH medium supplemented with 0.9% NaCl to achieve a final density of 1 × 10^6^ CFU mL^−1^. Aliquots of 50 μL of the bacterial suspension were added to 50 μL of the Ap9 solution serially diluted in sterile 0.1% BSA in a flat-bottom 96-well microplate. After incubation for 24 h at 37 °C with shaking at 950 rpm, initial MIC values were determined. For each subsequent daily passage, 2 μL of the culture from the well containing the highest sub-inhibitory peptide concentration was transferred into 1 mL of MH medium with 0.9% NaCl. From the obtained suspension, 50 μL was used to inoculate a new peptide dilution series. The selection procedure was continued up to 25 passages. After the final transfer, bacterial populations able to grow at the highest peptide concentration were plated on Ap9-free LB agar and subcultured for three days to stabilize the phenotype, after which the final MIC was determined. The experiment was carried out in 7 independent replicates.

Genomic DNA samples from Ap9-resistant isolates (EC3, EC5, EC7) were sequenced by Biospark LLC (Troitsk, Russia) on the Illumina NextSeq 550 platform (Illumina, San Diego, CA, USA). Read quality was assessed with FastQC, adapters and low-quality bases were trimmed with Trimmomatic, followed by a second quality check. Reads from the obtained resistant isolates were aligned to strain reference using BWA. To call actual variants, VarScan software was launched with a minimal reported variant frequency set to 0.9.

### 4.10. Determination of Growth Rate

Overnight cultures of *E. coli* were diluted in LB medium to 1 × 10^6^ CFU mL^−1^. Aliquots of 100 μL were dispensed into a flat-bottom 96-well microplate and incubated at 37 °C with shaking at 900 rpm. OD_570_ was measured using a microplate reader AF2200 (Eppendorf, Hamburg, Germany).

### 4.11. Biofilm Assay

Overnight cultures of *E. coli* were diluted 1:150 in LB medium. 100 µL aliquots were dispensed into a flat-bottom 96-well microplate and incubated at 32 °C with gentle shaking (120 rpm) for 24 h to allow biofilm formation. Next, the medium was removed, and the wells were washed three times with sterile water. Biofilms were stained with 0.1% crystal violet (CV, Sigma-Aldrich, St. Louis, MO, USA) for 40 min at 25 °C, rinsed to remove excess dye, and then treated with 30% acetic acid for 15 min at 25 °C to extract the bound CV. The solubilized dye was transferred to a new microplate, and absorption was measured at 570 nm using a microplate reader AF2200 (Eppendorf, Hamburg, Germany). Experiments were performed in triplicate.

### 4.12. Checkerboard Assay

Combinations of Ap9 with different antibiotics were tested by microdilution assay under the same conditions as described in Section 4.2. Two-fold serial dilutions of each agent were prepared in 96-well microplates, placing Ap9 by rows and an antibiotic by columns, with the highest concentrations located in opposite corners. Each plate included single-agent dilution rows/columns to verify MICs. After overnight incubation, the fractional inhibitory concentration index (FICI) was calculated as:

FICI = [A]/MIC_A_ + [B]/MIC_B_ (1)

where MIC_A_ and MIC_B_ are MICs of agents A and B, tested individually, and [A] and [B] are concentrations of A and B that inhibit growth when used in combination. A FICI ≤ 0.5 was taken to indicate synergy, 0.5 < FICI ≤ 1 was interpreted as an additive effect. Results are reported from three independent experiments.

### 4.13. Production of LptD/LptE Complex

Sequences of *lptD* and *lptE* were amplified from genomic DNA of *E. coli* MDR CI 1057 by PCR using the primers LPT-1/LPT-2 and LPT-3/LPT-4, respectively (Supplementary Table S1). The *lptD* amplicon was inserted into pTK expression vector (the pET-31/32 based plasmid) using XhoI and NdeI restriction sites (Supplementary Figure S1A). The *lptE* amplicon was cloned into pHybr vector using NdeI and BamHI restriction sites. pHybr is a hybrid plasmid assembled from pTK (the fragment amplified with primers pET-rev and pET-dir) and pLysS (the fragment amplified with primers pLys-rev and pLys-dir), as shown in Supplementary Figure S1A–C. Next, *lptE-his*_6_ and *lptD* were coexpressed from pHybr and pTK plasmid, respectively, in *E. coli* BL21 (DE3). After that, double transformants were selected on LB agar supplemented with chloramphenicol (34 μg mL^−1^) and ampicillin (100 μg mL^−1^). For generation of a fluorescent LptE fusion for microscale thermophoresis (MST) experiments, the GFP coding sequence was amplified from plasmid pJ23119-GFP using the primers LPT-5 and LPT-6 [43]. The PCR product was digested with BamHI and XhoI and ligated into the previously obtained pHybr vector carrying *lptE-his*_6_. As a result, a construct encoding LptE fused at its C-terminus via a flexible GSGS linker was obtained (Supplementary Figure S2B). The same two-step transformation strategy was applied to obtain *E. coli* BL21 (DE3) carrying pHybr-*lptE*-*gfp*-*his*_6_ and pTK-*lptD* (Supplementary Figure S2A–C).

10 mL of the overnight cultures of DE (pTK-*lptD* and pHybr-*lptE*-*his*_6_) and DEG (pTK-*lptD* and pHybr-*lptE*-*gfp*-*his*_6_) were transferred to 250 mL of LB medium containing 20 mmol glucose, ampicillin (100 μg mL^−1^), chloramphenicol (34 μg mL^−1^), 1 mmol MgSO_4_, and 1 mmol CaCl_2_ and were grown at 37 °C until OD_600_ reached values of 0.85 (DE, 4.5 h) and 0.4 (DEG, 8 h). Expression was induced by addition of 0.2 mmol IPTG and incubation at 22 °C for 17 h, resulting in final OD_600_ values of 6.2 (DE) and 4.6 (DEG). The DE and DEG complexes were purified by the same protocol described below. Cells were harvested (10,000 rpm, 15 min, 4 °C), and the pellet was resuspended in 6 mL of the buffer solution TBS-A (20 mmol Tris-HCl, 200 mmol NaCl, 8 mmol imidazole, pH 7.8) supplemented with 1 mmol PMSF. Lysis was performed by sonication on ice (10 s on/20 s off, 85% amplitude, total time of 6 min). Unbroken cells were removed by centrifugation at 5000 rpm, at 4 °C for 15 h, and membranes were collected by ultracentrifugation at 90,000 rpm, at 4 °C for 1 h. The membrane pellet was washed using 5 mL of TBS-A containing 0.5% N-lauroylsarcosine sodium salt (Solarbio, Beijing, China) and incubated at 4 °C for 1 h with gentle mixing, then centrifuged at 90,000 rpm, at 4 °C for 1 h to obtain outer membranes, which were then extracted in 13 mL of TBS-B (50 mmol Tris-HCl, 250 mmol NaCl, 10 mmol imidazole, pH 7.8) containing 1% lauryldimethylamine-N-oxide (LDAO, Anatrace, Maumee, OH, USA), lysozyme (100 μg mL^−1^) at 4 °C for 2 h with gentle mixing, and re-centrifuged at 90,000 rpm, at 4 °C for 1 h. The resulting supernatant was loaded onto 2 mL of Ni-NTA Sepharose (GE Healthcare, Chicago, IL, USA) pre-equilibrated with TBS-B buffer containing 1% LDAO. The resin was washed sequentially with TBS-B containing 1% LDAO, then TBS-B containing 0.1% LDAO, and finally TBS-B containing 1% n-Octyl-beta-D-glucopyranoside (OG, BLD Pharmatech, Shanghai, China). The complex was eluted with TBS-C (25 mmol Tris-HCl, 250 mmol NaCl, 250 mmol imidazole, pH 7.8) containing 1% OG. The fraction of DE and DEG complexes were analyzed by SDS-PAGE, Western blot method, and mass spectrometry.

### 4.14. MST Experiment

MST measurements were performed on a Monolith NT.115 instrument (NanoTemper Technologies, Munich, Germany) using standard capillaries (cat. тo. MO-K022). The *E. coli* LptD/LptE-GFP-His_6_ complex was kept at a constant concentration of 300 nmol in the buffer (25 mmol Tris-HCl, 250 mmol NaCl, 250 mmol imidazole, pH 7.8) containing 1% OG. 16-point serial dilutions of each peptide (Ap9, thanatin, tachyplesin I) were prepared in aqueous 0.1% BSA solution, resulting in concentrations from 40 μmol to 1 nmol. Equal volumes of ligand and protein solutions were mixed to a total volume of 20 μL. To remove potential aggregates, obtained mixtures were centrifuged for 5 min, at 10,000 rpm at room temperature. Measurements were carried out using 20% MST and 100% LED power settings. Each assay was performed in triplicate, and *K*_d_ were calculated with MO.Affinity Analysis v2.3 software.

### 4.15. NMR Spectroscopy

All NMR experiments for structural determination of the monomeric form of the peptide were performed on an Avance III 600 MHz spectrometer (Bruker Biospin, Rheinstetten, Germany) equipped with a TCI cryogenic probe. Spectra were recorded at 20, 30, and 40 °C. Data acquisition and processing were performed with topspin software v3.4 running on Linux workstation. 0.4 mmol Ap9 was dissolved in H_2_O/D_2_O (19:1) and adjusted to pH 4.8. ^1^H, ^13^C assignments were obtained using standard procedure, based on 2D TOCSY (40 and 80 ms mixing time), 2D NOESY (40, 80 ms and 150 ms mixing time), ^13^C-HSQC, and ^15^N-HSQC. After recording the set of spectra, Ap9 was lyophilized and dissolved in 100% D_2_O (CIL, Los Angeles, CA, USA) to measure the proton–deuterium exchange and record 2D NOESY (80 ms mixing time) and DQF-COSY spectra.

For calculating of the dimeric form of Ap9, NMR experiments were performed on an Avance II 700 MHz spectrometer (Bruker Biospin, Rheinstetten, Germany) equipped with a TCI cryogenic probe at 30 °C. 0.4 mmol Ap9 was dissolved in H_2_O/D_2_O (19:1, pH 4.8) and 0.4 mmol LPS was added. The resulting sample was sonicated for 30 min at 25 °C. ^1^H, ^13^C assignments were obtained using the same set of 2D NMR experiments described for the monomeric form.

### 4.16. Spatial Structure Calculations

Spatial structure calculations were performed using the simulated annealing/molecular dynamic protocol as implemented in the CYANA software package version 3.98.13 [44]. Upper interproton distance restraints were received by 1/r6 calibration of NOESY cross-peaks. Torsion angle restraints and stereospecific assignments were obtained from the J-couplings and NOE intensities. Hydrogen bonds and disulfide bonds were added at the final stage of calculations. The disulfide linkages were introduced based on NOE cross-peaks. Visual analysis of the calculated structures and figure drawings were performed using the PyMOL (Schrodinger, LLC) softwarev1.8. The structures of monomer and dimer were validated and deposited in the PDB database under accession codes 9XAO and 9XAP, respectively.

### 4.17. Peritonitis Model with Virulent E. coli 3421E/19

Male BALB/c mice with cyclophosphamide-induced immunosuppression (*n* = 5 per group, 20–22 g) were infected intraperitoneally with virulent *E. coli* 3421E/19 at 1 × 10^6^ CFU (0.5 mL/mouse). At 1 h post-infection, animals received a single intraperitoneal dose of Ap9 at concentrations from 1 to 16 mg kg^−1^. The treatment control group received polymyxin B at a dose of 5 mg kg^−1^ with the same route of administration. Mice were euthanized 6 h after infection. Bacterial burden in blood and peritoneal fluid was quantified (CFU mL^−1^) by plating on Endo agar supplemented with tetracycline at a concentration of 20 µg mL^−1^.

### 4.18. Peritonitis Model with P. aeruginosa PAO1

Male BALB/c mice (*n* = 5 per group, 18–20 g) were infected intraperitoneally with *P. aeruginosa* PAO1 at 1.6 × 10^5^ CFU (20 LD_50_) suspended in 0.9% saline containing 2.5% mucin. At 1 h post-infection, animals received a single intraperitoneal dose of Ap9 at concentrations from 1 to 16 mg kg^−1^. Treatment control group received polymyxin B at a dose of 5 mg kg^−1^ using the same route of administration. Mice were euthanized 6 h after infection. Bacterial burden in blood and peritoneal fluid was quantified (CFU mL^−1^) by plating on Pseudomonas cetrimide agar.

### 4.19. Survival Model with P. aeruginosa PAO1

Male and female BALB/c mice (*n* = 5 per group, 18–20 g) were infected intraperitoneally with *P. aeruginosa* PAO1 at 1.6 × 10^5^ CFU (20 LD_50_) suspended in 0.9% saline containing 2.5% mucin. Ap9 and polymyxin B were administered intraperitoneally at 15 mg kg^−1^ per dose at 1, 4, and 8 h post-infection. Mice were monitored for 72 h after infection and survival was recorded. Animals that died during the study were necropsied, and their spleens were examined by impression smears. After 72 h, surviving mice were euthanized, and blood and spleens were collected for detection of *P. aeruginosa* PAO1 by spleen impression smears and blood culture analysis.

### 4.20. Survival Model with Uropathogenic E. coli U10 (mcr-1-Mediated Resistance)

Male BALB/c mice (*n* = 45; 18–20 g) were randomized into 9 groups (8 treatment, 1 untreated control, *n* = 5 per group). All mice were challenged intraperitoneally with a lethal dose of *E. coli* U10 (1 × 10^7^ CFU in 2.5% mucin). Four groups received Ap9 at doses of 10, 5, 2.5, or 1.25 mg kg^−1^ at 1 h and 4 h post-challenge. Four other treatment groups received polymyxin B at the same single doses (10, 5, 2.5, 1.25 mg kg^−1^). All injections were administered at 0.5 mL per mouse. A control group did not receive treatment. Animals were observed for 7 days after challenge, and survival was recorded. Body weight was measured before dosing and on days 2 and 7. Spleens from deceased animals were examined for the presence of *E. coli* U10 by impression smears. At the end of the study, surviving animals were euthanized, necropsied, and spleen impression smears were prepared to detect the test strain.

### 4.21. Pharmacokinetic Study

SD rats (9–10 weeks, 275 ± 15 g) received Ap9 at a single intramuscular dose of 1 mg kg^−1^. Blood was collected from the lateral tail vein at 5, 10, 20, 40, 60, 180, 240, and 480 min (*n* = 3 per time point) into anticoagulant-coated tubes, and plasma was obtained by centrifugation at 1600 *g*, for 15 min, at 4 °C. Ap9 was extracted from plasma using SP Sepharose Fast Flow (GE Healthcare, Uppsala, Sweden) and analyzed by LC-MS on an Orbitrap Elite ETD mass spectrometer (Thermo Scientific, Waltham, MA, USA) coupled to the EASY-nLC 1000 (Thermo Scientific, Waltham, MA, USA). Reversed-phase separations were performed on laboratory-packed C18 columns (Phenomenex; 100 Å, 1.7 µm; 150–200 mm, 100 µm inner diameter) using a linear gradient of 5–55% acetonitrile at 300 nL min^−1^ over 50 min. Ionization was performed by nanoelectrospray method with spray voltage of 1800 V and capillary temperature of 200 °C. Quantification was performed using a calibration approach. Samples were prepared by adding known amounts of Ap9 into plasma obtained from untreated animals, followed by extraction and purification under the same conditions as described above. Ap9 plasma concentrations were determined relative to calibration samples and corrected for extraction recovery (72.3%).

### 4.22. Statistics

Statistical analysis was performed using GraphPad Prism 10 software. Data are shown as mean ± SD of at least three independent experiments. Statistical significance was defined as *p* values ≤ 0.05 using unpaired Student’s *t*-test.

## 5. Conclusions

In summary, Ap9 combines a potent antibacterial activity in vivo, a favorable tolerability, a broad therapeutic window, quite a low probability of resistance development, and synergy with conventional antibiotics, including last-resort agents. Importantly, the peptide Ap9 showed a rapid systemic absorption after intramuscular injection in rats, indicating a potential for the use in acute systemic infections and providing a basis for further pharmacokinetic studies in larger animal models. This makes Ap9 a promising candidate for the development of an alternative to polymyxin antibiotics for the treatment of multidrug-resistant Gram-negative infections.

## Figures and Tables

**Table 1:** Arenicin-like β-hairpin AMPs.

AMP	Sequence	Charge	GRAVY ^(a)^
**Abarenicin** (*A. pacifica*)	GYCFTACYMRNGVRICYRRCN	+4	−0.248
Ap9	GYCFTAC**AR**RNGVRICYRRCN	+5	−0.405
**Arenicin-3** (*A. marina*)	GFCWYVCVYRNGVRVCYRRCN	+4	−0.048
AA139	GFCWYVC**AR**RNG**A**RVCYRRCN	+5	−0.429
N1(NZ17074)	GFCW**N**VCVYRNGVRVC**H**RRCN	+4	−0.243
N6	GF**A**W**N**VCVYRNGVRVC**H**RR**A**N	+4	−0.310

^(a)^ Mean Kyte-Doolittle hydrophobicity index (GRAVY) was calculated using the Expasy ProtParam tool (https://web.expasy.org/protparam/, accessed on 1 October 2025). Cysteine residues are highlighted in yellow, amino acid substitutions in analogs are indicated in bold and underlined relative to the corresponding native peptides.

## Data Availability

The datasets generated or analyzed during this study are available from the corresponding author on reasonable request.

## Supplementary Materials

The following supporting information can be downloaded at: https://www.mdpi.com/article/10.3390/md24050154/s1. Table S1. Primers used in this study. Figure S1. Plasmid maps of pTK (**A**), pLysS (**B**) and pHybr (**C**). Figure S2. Construction of LptD/LptE-GFP-His6 complex. **A**) pTK-lptD map. **B**) pHybr-lptE-gfp-his6 plasmid map. **C**) PyMOL structural model of this complex (based on PDB: 4RHB). Table S2. Minimum inhibitory concentrations (MIC, μmol) of Ap9 and polymyxin B by action against *E. coli* strains in the MH broth supplemented with 0.9% NaCl. Figure S3. The structure of the *E. coli* LptD/LptE complex (PDB: 4RHB) was visualized in PyMOL. The K486Q substitution, located in a sterically unfavorable region for Ap9 binding, is highlighted in red. Figure S4. Production of LptD/LptE-GFP-His6 in *E. coli* BL21(DE3). Table S3. Statistics for the 10 best NMR structures of Ap9 in monomeric and dimeric forms. Figure S5. Ramachandran plots for monomeric (**A**) and dimeric (**B**) forms of Ap9. Figure S6. Overview of the NMR data that determine the secondary structure of monomeric (**A**) and dimeric forms of Ap9 (**B**). Figure S7. Comparison of the monomeric and dimeric structures of Ap9. Table S4. Acute single dose toxicity of Ap9 at 15 mg kg-1 in ICR mice. Figure S8. Evaluation of the outer membrane permeability of *E. coli* ML-35p induced by Ap9 using the chromogenic marker nitrocefin. Figure S9. Effect of Ap9 and the control AMP Bac7[1-35] at different concentrations on in vitro bacterial translation, measured as EGFP fluorescence in *E. coli* based cell-free system. Figure S10. Flow cytometric analysis of *E. coli* ML-35p cytoplasmic membrane depolarization after 1 h exposure to Ap9 using DiBAC4(3) and PI. Table S5. Key properties of Ap9 and arenicin-derived peptides.

## Abbreviations

The following abbreviations are used in this manuscript:

AMP	Antimicrobial peptide
LPS	Lipopolysaccharide
MIC	Minimum inhibitory concentration
MST	Microscale thermophoresis
PBS	Phosphate-buffered saline
BSA	Bovine serum albumin
SEM	Scanning electron microscopy
GFP	Green fluorescent protein
ONPG	O-nitrophenyl-β-D-galactopyranoside
BLM	Planar bilayer lipid membranes
FICI	Fractional inhibitory concentration index
