# Supplementary material for: Marine Antimicrobial Peptide as a Promising Alternative to Polymyxin B
Source: Mar Drugs. 2026 Apr 27;24(5):154. doi: 10.3390/md24050154 (PMC13208921; doi:10.3390/md24050154)
Supplement: Supplementary file 1 [file marinedrugs-24-00154-s001.zip › marinedrugs-4276919-supplementary.pdf]

Supporting Information

# Marine Antimicrobial Peptide as a Promising Alternative to Polymyxin B

**Victoria N. Safronova, Vladislav A. Lushpa, Victoria O. Shipunova, Marta V. Volovik, Kira L. Dobrochaeva, Roman N. Kruglikov, Ilia A. Bolosov, Dmitrii E. Dashevskii, Alexey V. Mishin, Oleg V. Batishchev, Olga V. Korobova, Alexander I. Borzilov, Gulsara A. Slashcheva, Igor A. Dyachenko, Eduard V. Bocharov, Pavel V. Panteleev and Tatiana V. Ovchinnikova**

**Supplementary Table S1.** Primers used in this study.

| **Primer Name** | **Sequence (5'→3')** |
|---|---|
| LPT-1 | GGAGATATACATATGAAAAAACGTATCCCCACTCTC |
| LPT-2 | CGCGGATCCTTACAAAGTGTTTTGATACGGCAGAATGT |
| LPT-3 | CGGGATCCGTTACCCAGCGTGGTGGAG |
| LPT-4 | GGAGATATACATATGCGATATCTGGCAACATTGTTG |
| pET-dir | CATACGATATAAAGATCTGCGGGATCTCGATCC |
| pET-rev | AAATGCCTGAGGATTCGCCAATCCGGATATAGTTCC |
| pLys-dir | GATTGGCGAATCCTCAGGCATTTGAGAAGCAC |
| pLys-rev | CCCGCAGATCTTTATATCGTATGGGGCTGACTTCAG |
| LPT-5 | CGGGATCCGGTAGCATGCGTAAAGGCGAAGAGCT |
| LPT-6 | CGCTCGAGACTACCTTTGTACAGTTCATCCATACCATGC |

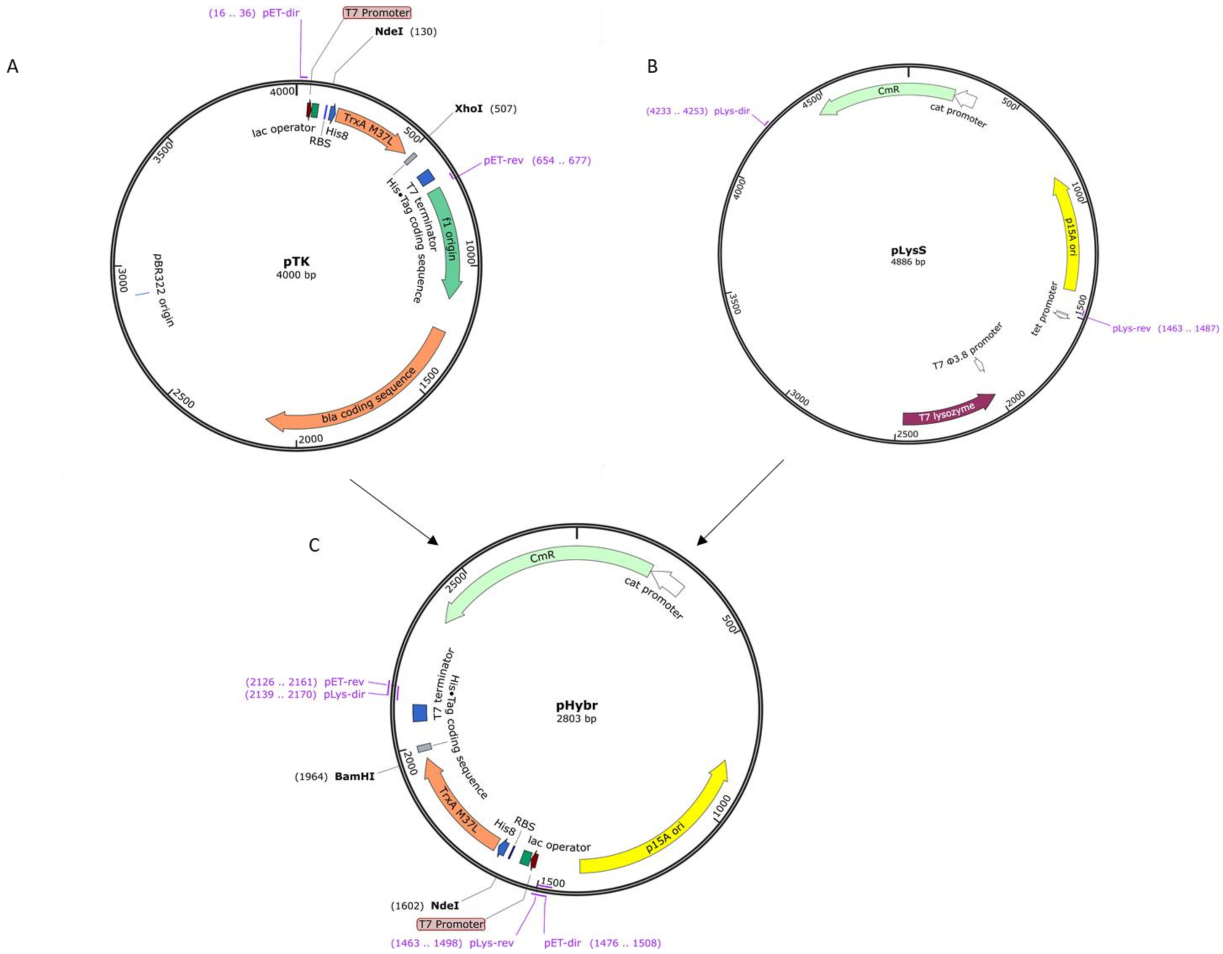


**Supplementary Figure S1.** Plasmid maps of pTK (**A**), pLysS (**B**) and pHybr (**C**).

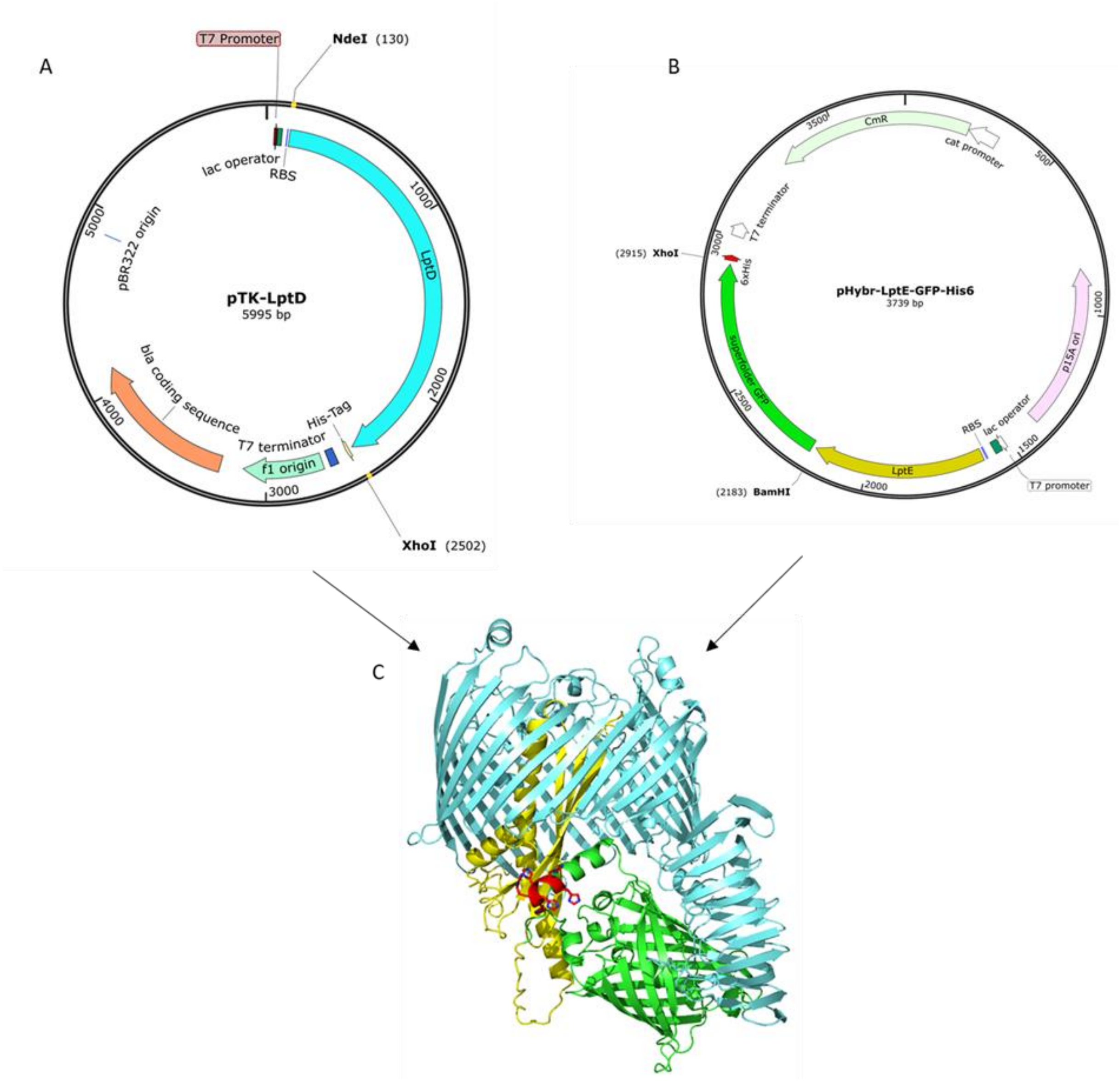


**Supplementary Figure S2.** Construction of LptD/LptE-GFP-His$_6$ complex. (**A**) pTK-*lptD* map. (**B**) pHybr-*lptE*-*gfp*-*his*$_6$ plasmid map. (**C**) PyMOL structural model of this complex (based on PDB: 4RHB).

**Supplementary Table S2.** Minimum inhibitory concentrations (MIC, μmol) of Ap9 and polymyxin B by action against *E. coli* strains in the MH broth supplemented with 0.9% NaCl.

| Strain | Ap9 | Polymyxin B |
|---|---|---|
| U10 (*mcr-1*) | 0.125 | 4 |
| P1[a)] (*bamA* (I130S), *sppA* (R371H), *spoT* (N118H), *rpoS* (Q304stop), *secA* (Q830stop), *pmrB* (V161G, S305R) | 0.125 | >32 |
| *P. mirabilis* XDR CI 3423 | 1 | >16 |

[a)]The strain P1 has been selected from *E. coli* MDR CI 1057 through polymyxin B resistance induction assay in our previous study. MIC values of Ap9 and polymyxin B for the wild type strain (ECwt) are shown in **Fig. 2E**.

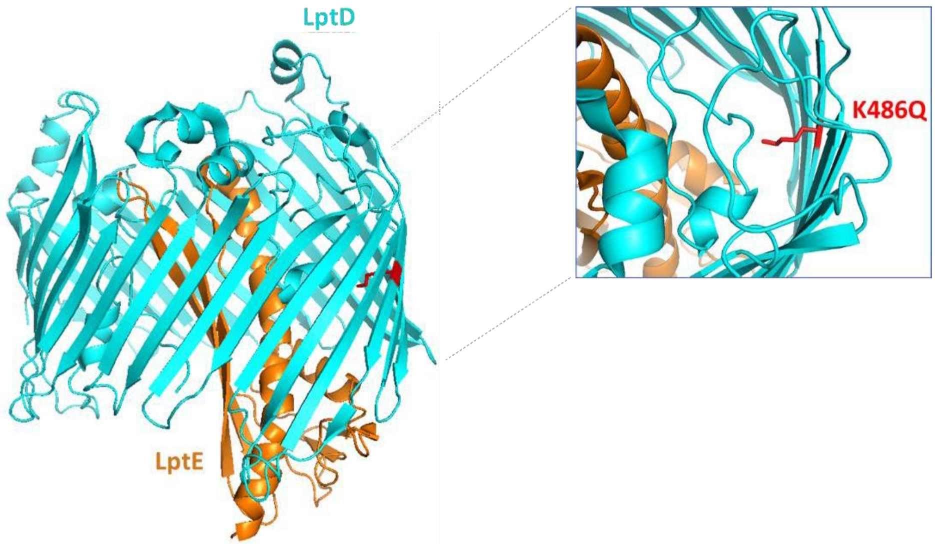


**Supplementary Figure S3.** The structure of the *E. coli* LptD/LptE complex (PDB: 4RHB) was visualized in PyMOL. The K486Q substitution, located in a sterically unfavorable region for Ap9 binding, is highlighted in red.

### SDS-PAGE and Western blot analysis

Fractions containing LptD/LptE-GFP-His6 or LptD/LptE-His6 complexes were mixed with the Laemmli sample buffer and separated by 12.5% SDS-PAGE at 200 V for 1 h. Equal amounts of the total protein were loaded per line, and gels were stained with Coomassie Brilliant Blue R-250. Bands of interest were excised from the gels and analyzed by mass spectrometry. For

immunodetection, proteins resolved by SDS-PAGE were transferred onto 0.2 µm nitrocellulose membranes (Bio-Rad, USA) using a wet transfer at 20 V for 1 h. Next, membranes were blocked with 5% non-fat dry milk in the TBST buffer (20 mmol of Tris-HCl, 150 mmol of NaCl, 0.1% Tween 20, pH 7.6) for 1 h at room temperature, followed by incubation with rabbit anti-6X His tag primary antibodies (Abcam, UK, 1:1000 dilution in TBST) for 1 h at room temperature. After three washes in TBST containing 5% milk, membranes were incubated with HRP-conjugated anti-rabbit IgG secondary antibodies (Sigma-Aldrich, USA, 1:25000 dilution in TBST containing 5% milk) for 1 h at room temperature. Finally, membranes were washed three times in the TBST buffer and incubated with Pico ECL reagent (Thermo Fisher Scientific, USA) according to the manufacturer's instructions, and visualized with a ChemiDoc imaging system (Bio-Rad, USA).

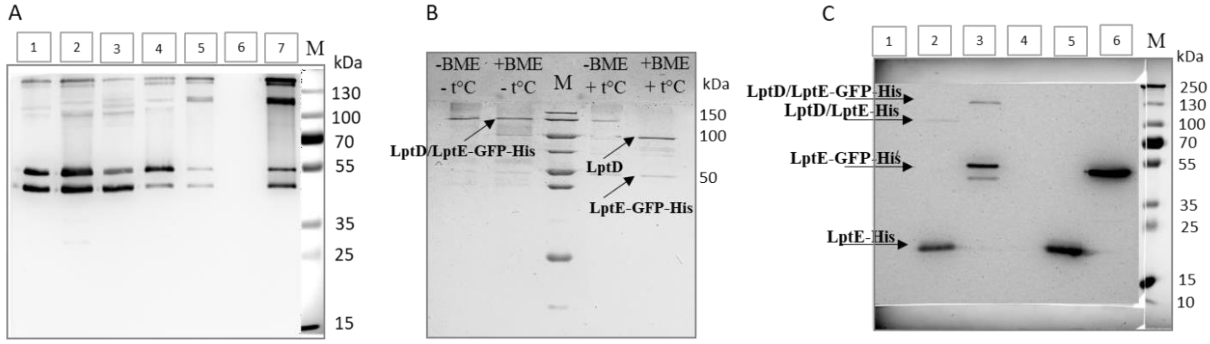


**Supplementary Figure S4.** Production of LptD/LptE-GFP-$His_6$ in *E. coli* BL21(DE3). (**A**) Western blot of fractions detected with anti-His antibodies: whole-cell lysate (1), pellet after SLS treatment (2), supernatant (3), pellet solubilized in LDAO (4), column load (5), flow-through (6), eluate (7). SDS-PAGE was performed without heating and in the presence of reducing agent (β-mercaptoethanol, BME). (**B**) SDS-PAGE (Coomassie staining) of purified complex under different conditions: with or without heating, and with or without BME. (**C**) Western blot analysis of LptD/LptE-GFP-$His_6$ (DEG) and LptD/LptE-$His_6$ (DE) expression after IPTG induction: before induction (1), DE cell lysate (2), DEG cell lysate (3), before induction (4), DE cell lysate (5), DEG cell lysate (6). Lanes 1 – 3 are non-heated samples + BME, 4 – 6 are heated samples + BME.

**Supplementary Table S3.** Statistics for the 10 best NMR structures of Ap9 in monomeric and dimeric forms.

| | **monomer** | **dimer** |
|---|---|---|
| **Parameter** | **Value** | |
| **Distance and angle restraints** | | |
| Total NOEs | | |
| intraresidual | 84 | 102 |
| interresidual | 110 | 230 |
| sequential($\|i-j\|=1$) | 36 | 52 |
| medium range($1<\|i-j\|\leq 4$) | 12 | 66 |
| long-range($\|i-j\|>4$) | 62 | 112 |
| Hydrogen bond restraints(upper/lower) | 15/15 | 48/48 |
| S-S bond restraints(upper/lower) | 6/6 | 12/12 |
| Angles | 32 | 66 |
| $\varphi$ | 32 | 66 |
| $\chi_1$ | 0 | 0 |
| **Total restraints/ per residue** | 268/21 | 518/42 |
| **Statistics of the obtained set of structures** | | |
| CYANA target function | 0.49 ± 0.05 | 2.67 ± 0.47 |
| Restraints violations | | |
| distance (>0.2Å) | 0 | 1 |
| angle (>5°) | 0 | 2 |
| RMSD(Å)<br>Elements of secondary structure: | | |
| backbone | 0.36 ± 0.16 | 0.47 ± 0.14 |
| all heavy atoms | 0.97 ± 0.20 | 1.33 ± 0.23 |
| **Ramachandran analysis** | | |
| residues in most favored regions (%) | 63.9 | 49.7 |
| residues in additional allowed regions (%) | 36.1 | 46.9 |
| residues in generously allowed regions (%) | 0 | 3.3 |
| residues in disallowed regions (%) | 0 | 0 |

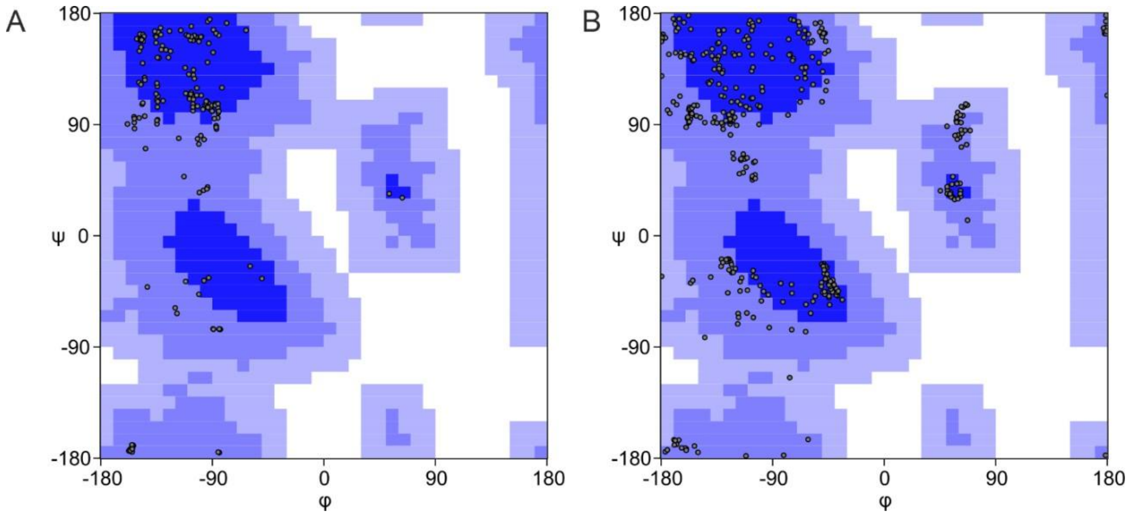


**Supplementary Figure S5.** Ramachandran plots for monomeric (**A**) and dimeric (**B**) forms of Ap9.

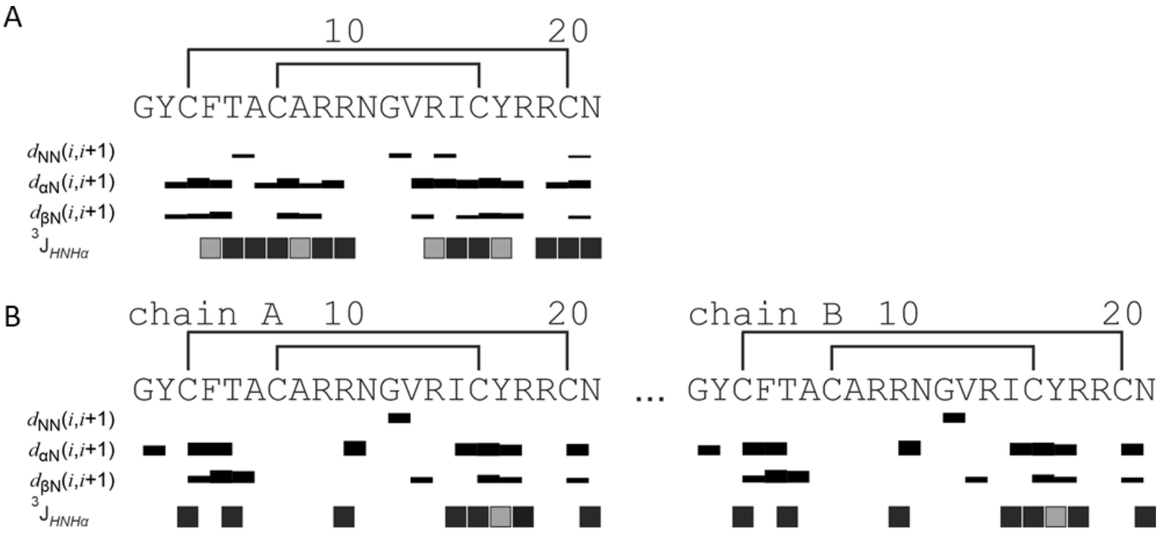


**Supplementary Figure S6.** Overview of the NMR data that determine the secondary structure of monomeric (**A**) and dimeric forms of Ap9 (**B**). The following parameters are listed from top to down: the peptide sequence with disulfide bonds (SS-bonds), NOE connectivities (dij) and 3JHNHα couplings. The bar widths represent the relative intensities of NOESY cross-peaks. Squares have three colors according to the value of J-coupling: black (>8 Hz), grey (6-8 Hz) and white (<6 Hz).

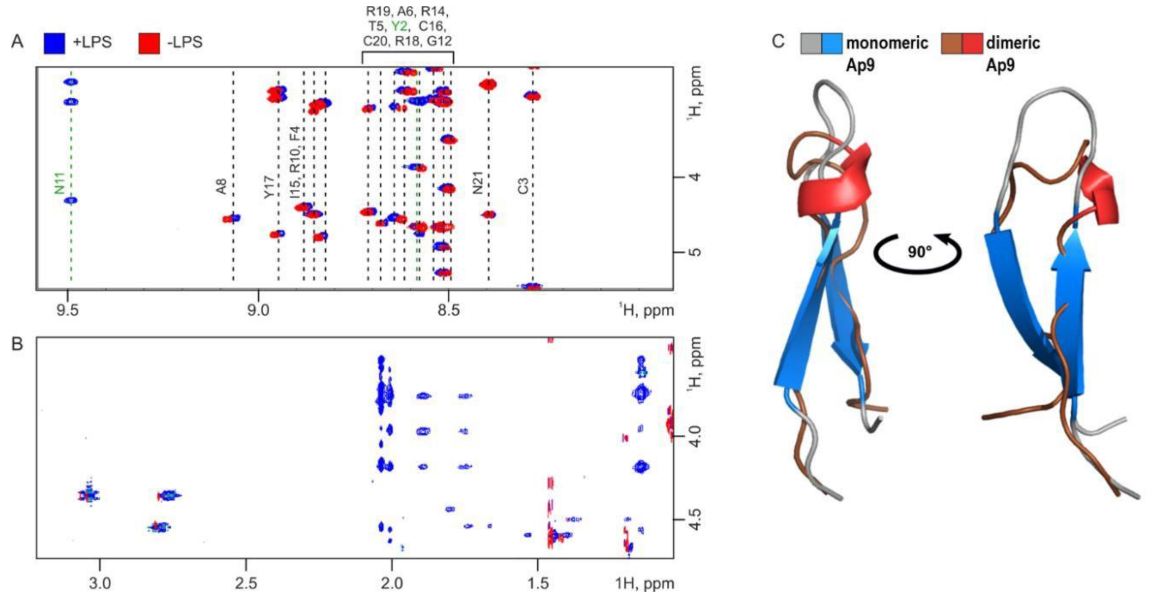


**Supplementary Figure S7.** Comparison of the monomeric and dimeric structures of Ap9. (**A**) Superposition of fragments of $^{1}$H,$^{1}$H-TOCSY spectra before (red) and after (blue) adding LPS. Chemical shifts of the fragment of the dimeric form undergoing changes are shown in green. (**B**) Superposition of fragments of $^{1}$H,$^{1}$H-NOESY NMR spectra Ap9 before and after adding LPS. New cross-peaks after LPS addition are shown. (**C**) Superposition of spatial structures of monomeric and dimeric forms of Ap9. The monomeric form is colored blue (β-sheet) and gray (loop), the dimeric form is colored red (helix) and brown (loop).

## Acute toxicity study

Female ICR mice (10 weeks, 25-30 g) received Ap9 or AA139 intravenously via the lateral tail vein at doses of 15, 30 or 45 mg $kg^{-1}$ in a total volume of 10 mL kg-1, administered as two equal boluses within 30 min of each other (n = 3 per dose, vehicle n = 5). Animals were observed twice daily for mortality and moribundity, and a detailed examination was performed immediately after administration and then on days 2, 7 and 14. Locomotor activity was assessed at 1 h post-dose using an OPTO-VARIMEX with Auto-Trek Version 4.2 (Columbus Instruments, USA). Body weight was measured prior to administration and on days 2, 7 and 14. On day 14, mice were euthanized in a CO2 chamber and dissected. A gross examination of internal organs was performed. Organ weights and macroscopic findings were recorded, and tissue were collected for

histological analysis. Fixed tissues were dehydrated and embedded in paraffin. Sections were stained with hematoxylin and eosin and examined by light microscopy.

**Supplementary Table S4.** Acute single dose toxicity of Ap9 at 15 mg $kg^{-1}$ in ICR mice.

| № | Category | Measure/ Organ | Units | 0.9% NaCl (n = 5) | Ap9 (n = 3) | Summary |
|---|---|---|---|---|---|---|
| 1 | Behaviour (3 min) | Distance traveled | m | 38.4 ± 9.5 | 36.0 ± 4.9 | No change vs control |
| 2 | Behaviour (3 min) | Rearing | n | 19.4 ± 4.4 | 28.0 ± 9.0 | No change vs control |
| 3 | Behaviour (3 min) | Resting time | s | 49.9 ± 13.3 | 54.5 ± 8.8 | No change vs control |
| 4 | Behaviour (3 min) | Movement time | s | 130.1 ± 13.3 | 125.5 ± 8.8 | No change vs control |
| 5 | Relative organ weight | Brain | g | 0.525 ± 0.01 | 0.523 ± 0.036 | No change vs control |
| 6 | Relative organ weight | Heart | g | 0.150 ± 0.007 | 0.152 ± 0.014 | No change vs control |
| 7 | Relative organ weight | Kidneys | g | 0.397 ± 0.016 | 0.389 ± 0.059 | No change vs control |
| 8 | Relative organ weight | Liver | g | 1.51 ± 0.08 | 1.33 ± 0.16 | No change vs control |
| 9 | Relative organ weight | Lungs | g | 0.193 ± 0.015 | 0.187 ± 0.015 | No change vs control |
| 10 | Relative organ weight | Spleen | g | 0.102 ± 0.021 | 0.095 ± 0.017 | No change vs control |
| 11 | Histopathology | Brain | --- | No lesions (0/5) | No lesions (0/3) | No pathological changes |
| 12 | Histopathology | Heart | --- | No lesions (0/5) | No lesions (0/3) | No pathological changes |

| | | | | | | |
|---|---|---|---|---|---|---|
| 13 | Histopathology | Lungs | --- | No lesions (0/5) | No lesions (0/3) | No pathological changes |
| 14 | Histopathology | Stomach | --- | No lesions (0/5) | No lesions (0/3) | No pathological changes |
| 15 | Histopathology | Kidneys | --- | Retention cysts (mild, 1/5; minimal, 3/5); Proximal convoluted tubule epithelial dystrophy (mild, 1/5) | Proximal convoluted tubule epithelial dystrophy (mild, 3/3) | No pathological changes |
| 16 | Histopathology | Liver | --- | Karyomegaly (minimal, 2/5) Single-cell apoptosis (minimal, 1/5) Macrosteatosis foci (mild, 1/5) | Single-cell apoptosis (minimal, 2/3; mild 1/3) | No pathological changes |
| 17 | Histopathology | Spleen | --- | Megakaryocytic extramedullary hematopoiesis (minimal, 2/5; moderate 3/5) | No lesions (0/3) | No pathological changes |
| 18 | Histopathology | Tail (injection site) | --- | No lesions (0/5) | No lesions (0/3) | No pathological changes |

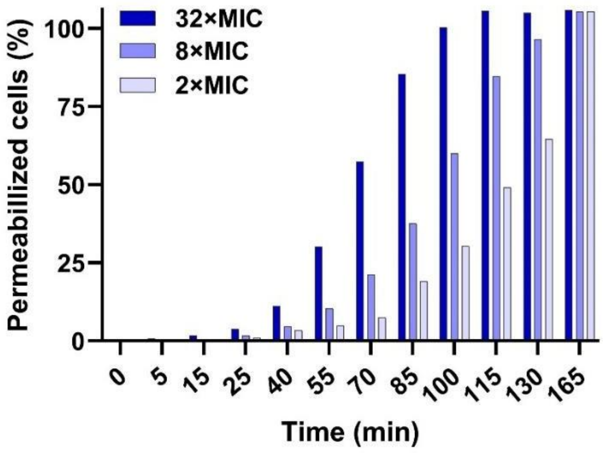


**Supplementary Figure S8.** Evaluation of the outer membrane permeability of *E. coli* ML-35p induced by Ap9 using the chromogenic marker nitrocefin. Melittin at 4 μmol served as a positive control.

**Inhibition of protein biosynthesis *in vitro***

The ability of the peptides to inhibit bacterial translation was evaluated using a cell-free protein synthesis (CFPS) system producing enhanced green flourescent protein (EGFP) under the control of the T7 promoter, as described previously with minor modifications [43]. Briefly, peptides were serially dilluted in 17 μL of 0.1% BSA using black polypropelene 96-well plates. Subsequenly, 33 μL of the CFPS reaction mixture was added to each well. The reaction was carried out for 2 h at 30 °C with shaking at 800 rpm using a plate shaker (Biosan, Latvia). Fluorescence of synthesized EGFP was measured using the AF2200 microplate reader ($\lambda_{Exc}$ = 485 nm, $\lambda_{Em}$ = 535 nm). Fluorescence in the of the peptide was set to 100%. Data represent mean values from two independent experiments.

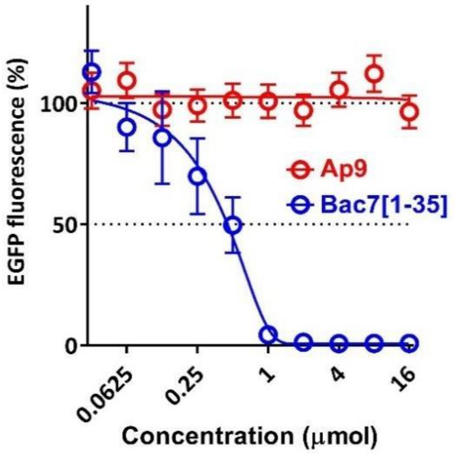


**Supplementary Figure S9.** Effect of Ap9 and the control AMP Bac7[1-35] at different concentrations on *in vitro* bacterial translation, measured as EGFP fluorescence in *E. coli* based cell-free system.

**Analysis of cytoplasmic membrane depolarisation**

*E. coli* ML-35p was grown in LB medium at 37 °C until an optical density at $OD_{600}$ reached 0.5, after which the cells were diluted 10-fold in PBS (pH 7.4) supplemented with 20 mmol glucose and 0.05% BSA. The resulting suspension was incubated with the tested peptide at 37 °C, 900 rpm for 1 h. Next, PI and DiBAC4(3) were added to final concentrations of 5 µg mL—-1 and 2 µmol respectively, and incubated in the dark at 37 °C for 15 min. The cells were harvested by centrifugation at 5000 g for 5 min, washed twice with cold PBS, resuspended in 1 mL of PBS and analysed by flow cytometry using a NovoCyte 2060R instrument (ACEA Biosciences Inc). The data obtained were processed using NovoExpress software version 1.2.4 (ACEA Biosciences).

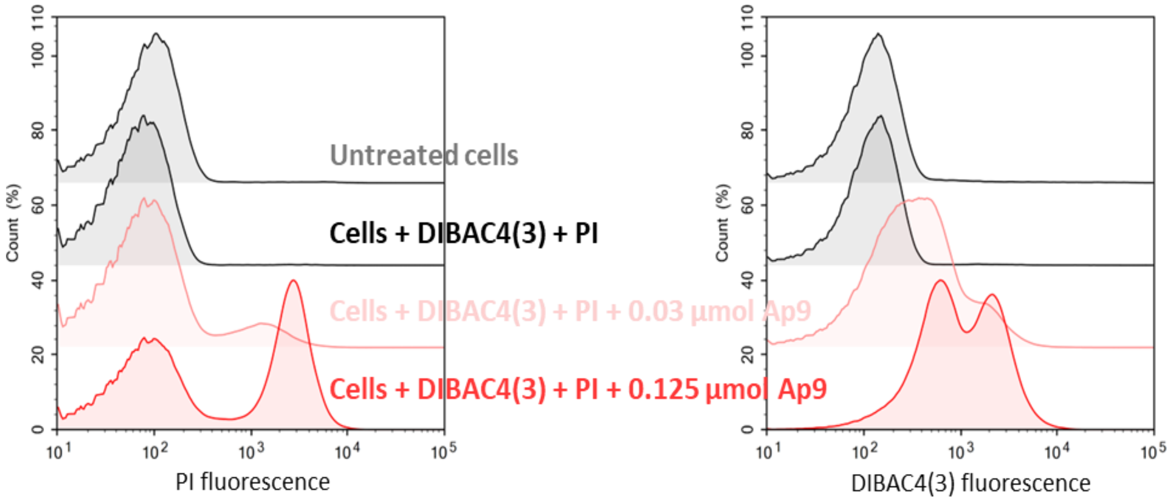


**Supplementary Figure S10.** Flow cytometric analysis of *E. coli* ML-35p cytoplasmic membrane depolarization after 1 h exposure to Ap9 using DiBAC4(3) and PI.

**Supplementary Table S5.** Key properties of Ap9 and arenicin-derived peptides.

| Parameter | Ap9 | AA139 | N6NH2 | N6 |
|---|---|---|---|---|
| MIC against *E. coli* | 0.125µmol (*E. coli* ATCC25922) | 0.25 µmol (*E. coli* ATCC25922) | 0.646 µmol (*E. coli* CVCC25922*) | 1.61 µmol (*E. coli* CVCC25922*) |
| Resistance-associated mutations | Mutations identified after 30 serial passages (7 independent lines), including *serS, mlaD, mlaA lptD* | Not defined for AA139. For arenicin-3: mutation in *mlaC* after 20 serial passages (1 line) | Mutations identified after 30 serial passages (3 independent lines), including k*psD, mlaA, gltC, mukB, bamA, hemE, rpoC, pldA, wzyE, ompR, rppH pheS, rcsF, aceE, rpoB, trkH,* | No resistant *E. coli* mutants obtained after 18 serial passages |
| Survival in murine *E. coli* infection models | 100% survival (*E. coli* ATCC 25922 infection, i.p., 2x10 mg $kg^{-1}$) 100% survival at 2x5 mg $kg^{-1}$ and 80% at 2x2.5 mg $kg^{-1}$ (*E. coli* U10, *mcr*-$1^{+}$) | 87.5% survival (*E. coli* ATCC 25922 infection, i.p., 2x10 mg $kg^{-1}$) | Not reported | 100% survival at 5 mg $kg^{-1}$; 66.7% at 2.5 mg $kg^{-1}$) (*E. coli* CVCC1515 infection) |
| *In vivo* efficacy (reduction of bacterial burden) | ~6-log reduction in peritoneal fluid and ~4–5-log reduction in blood in peritonitis model (*E. coli* 3421E/19) at 1 mg $kg^{-1}$ | ~3-log reduction in peritoneal fluid and ~4-log reduction in blood in peritonitis model (MDR *E. coli* AID#172) at 3.75 mg $kg^{-1}$ | Not reported | Not reported |
| Acute toxicity in mice | No histopathologica l alterations at 15 mg $kg^{-1}$ (i.v.) | Mild-to-moderate histological changes in the liver and | Not reported | Not reported |
| | | kidneys at 15 mg $kg^{-1}$ (i.v.) | | |

| | | | | |
|---|---|---|---|---|
| Half-life | ~2 h (rat, i.m.) | ~2–4 h (monkey, mouse, minipig, infusion, bolus) | Not reported | Not reported |

**E. coli* CVCC 25922 corresponds to the commonly used reference strain ATCC 25922, although minor laboratory-specific variations cannot be excluded.
